# Block and Statistical Copolymers of Methacrylate Monomers with Dimethylamino and Diisopropylamino Groups on the Side Chains: Synthesis, Chemical Modification and Self-Assembly in Aqueous Media

**DOI:** 10.3390/polym16091284

**Published:** 2024-05-03

**Authors:** Kalliopi Makri, Stergios Pispas

**Affiliations:** Theoretical and Physical Chemistry Institute, National Hellenic Research Foundation, 48 Vassileos Constantinou Avenue, 11635 Athens, Greece; mkr.kalliopi@gmail.com

**Keywords:** amphiphilic diblock copolymers, amphiphilic random copolymers, RAFT polymerization, polyelectrolytes, polyampholytes, micelles, aggregates

## Abstract

The synthesis of amphiphilic diblock and statistical (random) copolymers of poly(dimethylamino ethyl methacrylate) and poly((2-(diisopropylamino) ethyl methacrylate) using the reversible addition–fragmentation chain transfer polymerization technique (RAFT polymerization) is reported. The precursor copolymers were chemically modified to create derivative copolymers of polyelectrolyte and polyampholyte nature with novel solution properties. Moreover, their molecular and physicochemical characteristics, as well as their self-assembly in aqueous media as a function of molecular architecture and composition, are investigated by using size exclusion chromatography, spectroscopic characterization techniques and light scattering techniques. Furthermore, the behavior and properties of the obtained micelles and aggregates were studied, depending on the pH, temperature and ionic strength of the aqueous solutions. The response of the systems to changes in these parameters shows interesting behavior and new properties that are useful for their utilization as nanocarriers of pharmaceutical compounds.

## 1. Introduction

Over recent decades, there has been an accelerating evolution in the polymer field, especially in new techniques of polymerization and their ability to create a vast number of polymers with a variety of properties. Reversible addition fragmentation chain transfer polymerization (RAFT) is a polymerization technique which provides vitality to the synthesis process and control over living free radical characteristics [1]. Through controlling the agents in the RAFT synthetic process, well-defined polymers of various architectures, composition, and molecular weights can be synthesized, having essentially low molecular mass dispersity and high end-group functionality [2]. The ability of RAFT polymerization to tolerate a wide variety of monomers, their functionalities, the polymerization medium, and experimental conditions provides significant advantages over other controlled-living free radical polymerization processes. RAFT polymerization provides all the characteristics of controlled polymerizations, including predetermined molecular weights, narrow molecular weight distributions, and, most importantly, the reactivation of polymer chains for the synthesis of linear block copolymers, linear statistical copolymers, or other macromolecular architectures [3,4,5,6].

The impact of polymers’ amphiphilicity on the way they self-assemble in aqueous solutions should also be noted. Amphiphilic block and statistical copolymers consisting of two distinct segments, one hydrophilic and one hydrophobic, have been thoroughly studied due to their ability to self-assemble in aqueous media [7,8]. Generally, block copolymers have been studied for their self-assembly in aqueous solutions, forming a well-defined morphology, known as micelles [9]. Less research, but extremely important research, has been conducted for statistical (or random) copolymers for the same purpose. When in aqueous solutions, random copolymers form aggregates, which are not as well-defined as micelles of block copolymers, since the comonomers are randomly dispersed along the polymer chain [10]. However, their nanostructures could be beneficial regarding drug encapsulation or release. This ability of self-assembly makes polymers a great means for biomedical applications, such as gene delivery, drug delivery, or macromolecules delivery [11]. According to the monomeric units of polymers, they tend to respond to external factors, such as changes in pH and temperature or even to the ionic strength of the solution. Various studies have indicated that in a particular temperature range, thermo-responsive polymers undergo a volume phase transition that modifies their solubility [12]. It is accepted that this is a result of the hydration and dehydration of the chains. In brief, polymers interact with water molecules in the aqueous solution through hydrogen bonds, but with increasing temperature, there is a critical point (LCST) where hydrogen bonds break and polymers form globular aggregates. On the other hand, when this transition of solubility occurs with decreasing temperature, then the presence of UCST characterizes the polymer/aqueous system [13]. Nevertheless, most studies are about polymeric systems with LCST [14]. Similar to thermo-responsive polymers, pH-responsive polymers experience structural and property changes [15]. An additional important factor is the presence of salt in a polymer solution, which could lead to either salting-in or salting-out effects for polymer chains, mostly based on polymer and salt concentrations and the nature of the salt species [16].

In this study, the synthesis, chemical modification, and characterization of novel amphiphilic block and statistical copolymers consisting of the hydrophilic poly(dimethylamino ethyl methacrylate) and the potentially hydrophobic poly((2-(diisopropylamino) ethyl methacrylate) using the reversible addition–fragmentation chain transfer polymerization technique are reported. Moreover, CH_3_I and 1,3-cyclopropanesultone were used to quaternize the tertiary amine groups of the PDMAEMA block of both block and statistical copolymers in order to modify them into cationic and zwitterionic amphiphilic block and statistical polyelectrolytes, respectively. In particular, the quaternization of the PDMAEMA tertiary amine group provides the copolymer with permanent positive charges and improves the solubility in water [17]. PDMAEMA’s cationic charge allows complexation with negatively charged biomolecules, including proteins, nucleic acids (DNAs, RNAs), and so forth. Consequently, in gene therapy, these copolymers can be used as gene transfer agents. For drug delivery applications, the sulfobetainization of PDMAEMA creates zwitterionic materials which are more biocompatible [18].

PDMAEMA is among the most interesting and thoroughly researched stimuli-responsive polymers. PDMAEMA has tertiary amine side groups and is classified as a basic methacrylate polymer. It is a biocompatible weak cationic polyelectrolyte with interesting properties that responds to a wide range of factors, including pH, temperature, and ionic strength. Beyond its reaction to external stimuli, the modification of the tertiary amine to quaternary provides additional properties, such as the complexation of its positively charged chains with negatively charged macromolecules (nucleic acids, proteins). The second block of the copolymer is PDIPAEMA. It contains side chain tertiary amino groups, which are sterically hindered by two isopropyl groups. Due to these substituents, PDIPAEMA is hydrophobic, and in combination with PDMAEMA, they could form interesting supramolecular structures in aqueous solutions that are worthwhile to study [19]. The different substitutions of the amino side group are the fundamental differences between the two monomers/copolymers used in this study.

## 2. Materials and Methods

### 2.1. Materials

The monomers (dimethylamino)ethyl methacrylate (DMAEMA) and 2-(diisopropylamino)ethyl methacrylate (DIPAEMA) were purified, passing through a column packed with inhibitor-removing resins 311,340 and 311,332 from Sigma-Aldrich (Athens, Greece). 2,2-azobis(isobutyronitrile) (AIBN) was recrystallized from methanol and was used as the radical initiator. 1,4-dioxane (99.8% pure) was chosen as the solvent of the reaction and was dried using molecular sieves. 4-cyano-4-(phenylcarbonothioylthio) pentanoic acid (CPD) was the chain transfer agent of the reaction, and n-hexane, tetrahydrofuran (THF), pyrene, deuterated chloroform (CDCl_3_), deuterated water (D_2_O), CH_3_I and 1,3-cyclopropanesultone were used as received (all from Sigma-Aldrich). Dialysis tubing membranes from the regenerated cellulose of MWCO 3500 and with a diameter of 22 mm were purchased from SERVA, Heidelberg, Germany.

### 2.2. Characterization Methods

#### 2.2.1. Size Exclusion Chromatography (SEC)

Using size exclusion chromatography, the molecular mass and mass distributions of the synthesized block and statistical copolymers were determined. A Waters SEC set-up was used, consisting of an isocratic Water 1515 pump, a set of three µ-Styragel mixed-composition separation columns (with pore range 10^2^–10^6^ Å), a Waters 2414 refractive index detector (at 40 °C) and Breeze software (Breeze v2.0 Waters Corporation, Milford, MA, USA) for SEC set-up control. Tetrahydrofuran was used as the solvent, which contained 5% *v*/*v* triethylamine, at a flow rate of 1 mL/min at 30 °C. The calibration of the system was performed using polystyrene standards with a narrow distribution of molecular weights and average molecular weights of 1200–92,900 g/mL. The polymer samples were dissolved in tetrahydrofuran at concentrations in the range of 1–4 mg/mL.

#### 2.2.2. Proton Nuclear Magnetic Resonance Spectroscopy (^1^H-NMR)

^1^H-NMR spectra were obtained using a Bruker AC 300 FT-NMR spectrometer at 30 °C. Chemical shifts are shown in ppm in reference to tetramethyl silane (TMS). Regarding sample preparation, 10 mg of copolymer were dissolved in 0.7 mL deuterated chloroform (CDCl_3_) or deuterated water (D_2_O) and then the solution was introduced to NMR tubes. The NMR spectrum analysis was performed using MestReNova software (VNMRJ 2.2C, Varian, Palo Alto, CA, USA) from MestRelabs.

#### 2.2.3. Attenuated Total Reflectance-Fourier Transform Infrared Spectroscopy (ATR-FTIR)

Measurements in the near-infrared region (550–4000 cm^−1^) were performed using a Fourier transform spectrometer (Equinox 55 from Bruker Optics, Ettlingen, Germany) equipped with a single reflection ATR diamond (Dura-Samp1IR II from SensIR Technologies, Danbury, CT, USA) in a room temperature environment and in solid phase.

#### 2.2.4. Fluorescence Spectroscopy (FS)

The determination of critical micellization concentration (CMC) or critical aggregatation concentration (CAC) was achieved using a NanoLog fluorimeter (Horiba Jobin Yvon, Palaiseau, France) equipped with a laser diode as the excitation source (NanoLED, 440 nm, range of pulse 100 ps) and a UV detector TBX-PMT series (250–850 nm) from Horiba Jobin Yvon. Solutions of each polymer were prepared in a range of concentrations between 10^−3^ and 10^−8^ mg/mL, and pyrene solution in acetone, which was used for tracing, was added to all samples at a ratio of 1 μL pyrene solution/1 mL copolymer solution. The samples were kept at rest for 24 h to ensure the encapsulation of pyrene into the hydrophobic domains of the polymer aggregates and the evaporation of acetone. The excitation wavelength used for the measurements was 335 nm. Emission spectra were recorded in the spectral range of 355–640 nm. The ratio I_1_/I_3_, or, in other words, the ratio of intensities of the first and third vibronic peaks in pyrene fluorescence spectra, was utilized in order to access the hydrophobicity of the pyrene environment within the polymer aqueous solutions.

#### 2.2.5. Dynamic Light Scattering (DLS)

Dynamic Light Scattering studies were carried out with an ALV/GS-3 compact goniometer system (ALV GmbH, Hessen, Germany), equipped with a JDS Uniphase 22 mW vertically polarized He–Ne laser, operating at a 632.8 nm wavelength. The system is also equipped with an ALV/LSE-5003 light-scattering electronics unit used for stepper motor drive and limit switch control and an ALV-5000/EPP multi-τ correlator including 288 channels. The intensity of scattered light and the correlation functions were measured at 45°, 90° and 135° depending on temperature. The results presented below are measurements taken at 90°. Correlation functions were recorded five times and analyzed with the cumulants method and the CONTIN algorithm, which provides the distributions for the apparent hydrodynamic radius using an inverse Laplace transform of the autocorrelation function and through the Stokes–Einstein relation.

Measurements were typically taken in aqueous polymer solutions at a concentration of 10^−3^ g/mL in order for the laser beam to pass through the sample at the temperature where the solutions’ turbidity significantly increased. Additionally, when it was judged necessary, samples were diluted 1:5 since the laser beam could not pass through them at 25 °C. Prior to measurement, all solutions were filtered through a hydrophilic PVDF filter with a porosity of 0.45 µm.

#### 2.2.6. Electrophoretic Light Scattering (ELS)

Zeta potential values, which are directly related to the surface charge of polymer particles, were measured by electrophoretic light-scattering experiments conducted on a Nano Zeta Sizer instrument from Malvern, which is equipped with a 4 mW He–Ne laser, operating at 633 nm and a scattering angle of 173°. The LDV (Laser Doppler Velocimetry) method and the Smoluchowski approach were used in the electrokinetic measurements to determine the mobility and ζ_p_ values of the colloids. Experiments were conducted at 25 °C. The ζ_p_ values that are reported are the average of 100 measurements and the concentrations used for these experiments were the same as those in dynamic light scattering.

### 2.3. Polymer Synthesis via RAFT Polymerization

In this study, amphiphilic block and statistical copolymers were synthesized. The former consists of one block of poly((dimethylamino)ethyl methacrylate) (PDMAEMA) and one block of poly(2-(diisopropylamino)ethyl methacrylate) (PDIPAEMA), while the latter copolymers include the same monomers in random arrangement.

#### 2.3.1. Synthesis of PDMAEMA Homopolymer

The synthesis of PDMAEMA homopolymer was achieved via RAFT polymerization. Purified monomer (2.14 mL), AIBN (3.2 mg) and CPD (80.7 mg) were placed in a 25 mL round-bottom flask and were all dissolved in 1,4-dioxane (8 mL). The CPD–AIBN ratio was adjusted to 10:1 (mol). The flask was sealed with a rubber septum, and the deoxygenation of the polymeric solution was achieved via nitrogen bubbling for 20 min. Then, the flask was placed in an oil bath at 70 °C while stirring and left there for 18 h. Afterward, the flask was placed directly at −8 °C for 20 min and finally exposed to air for the termination of polymerization. Finally, the homopolymer was precipitated in excess hexane, and the final product was collected and placed in a vacuum oven for 48 h.

#### 2.3.2. Synthesis of PDMAEMA-b-PDIPAEMA Copolymers

Two block copolymers were synthesized (B1 and B2) via RAFT polymerization, with different ratios of the two blocks. PDMAEMA homopolymer was used as the macro-CTA for the second block polymerization. Purified monomer (DIPAEMA) (0.88 mL for B1 and 1.16 mL for B2), macro-CTA (0.4 g for B1 and 0.23 g for B2) and AIBN (1.6 mg for B1 and 0.89 mg for B2) were placed in a round-bottom flask, and 1,4-dioxane (6 mL for B1 and 4.95 mL for B2) was used as the solvent. The macro-CTA–AIBN ratio was adjusted to 5:1 (mol). The polymerization process was similar to what was described in Section 2.3.1. However, the isolation of the final product was achieved with the dialysis method. Deionized water was added to the polymerization solution (2:1 volume ratio), and this new solution was transferred to dialysis tubing. The tubing was sealed and placed in a beaker containing deionized water. Due to the pore size of the membrane, monomers and low molecular weight polymers can be transferred to the outer aqueous phase. After 48 h and six changes in the external aqueous phase, the solution inside the membrane was collected. Then, the solvent of the mixture was evaporated using a rotary evaporator, and the polymer was collected and placed in the vacuum oven for 48 h to dry.

#### 2.3.3. Synthesis of P(DMAEMA-co-DIPAEMA) Copolymers

Two statistical (random) copolymers were synthesized (Co1 and Co2) with different ratios of monomers. The RAFT polymerization procedure is similar to that described above (Section 2.3.1). The only difference is that this polymerization took place in one step. In other words, purified monomers of DMAEMA (1.07 mL for Co1 and 0.64 mL for Co2) and DIPAEMA (1.11 mL for Co1 and 1.56 mL for Co2), AIBN (3.3 mg for both copolymers), CPD (80.7 mg for both Co1 and Co2) and 1,4-dioxane (8 mL for both Co1 and Co2) were added to a 25 mL round-bottom flask. The CPD–AIBN ratio was adjusted to 10:1 (mol). The rest of the conditions of the polymerization remained the same. In the last step, the purification and isolation of the copolymers was achieved via the dialysis method, as reported in Section 2.3.2. 

### 2.4. Synthesis of Chemically Modified Copolymers

Block and random copolymers were chemically modified, so that the tertiary amine was converted to quaternary using different quaternization reagents. The aim of this conversion was to create polyelectrolytes, with different properties from the corresponding precursor copolymers.

#### 2.4.1. Synthesis of QPDMAEMA-b-PDIPAEMA and QP(DMAEMA-co-DIPAEMA)

The hydrophilic block/segment of PDMAEMA-b-PDIPAEMA and P(DMAEMA-co-DIPAEMA) copolymers is the one which contains DMAEMA, a weak cationic polyelectrolyte. The modification of the tertiary amine group to a quaternary ammonium salt, which is a strong cationic polyelectrolyte, was performed with the use of CH_3_I. The reaction on the amino group of PDMAEMA was carried out in a round-bottom flask containing a dilute solution (2% *w*/*v*) of the copolymer (0.11 g) in tetrahydrofuran (THF) (10 mL), and then under moderate stirring, the required amount of CH_3_I (41.2 mL) was added in a molar ratio to amine groups of 1:1 to achieve 100% quaternization. The reaction lasted for 24 h under constant stirring and at room temperature. The final product was collected after THF evaporation in a rotary evaporator and placed in a vacuum oven for 48 h for drying.

#### 2.4.2. Synthesis of SPDMAEMA-b-PDIPAEMA and SP(DMAEMA-co-DIPAEMA)

The modification of the tertiary amino group of DMAEMA to a sulfobetaine group was achieved via the reaction of the polymer with the proper amount of 1,3-cyclopropanesultone. A dilute solution (2% *w*/*v*) of polymer (0.11 g) in THF (10 mL) was prepared and then placed in a round-bottom flask. The required amount of 1,3-cyclopropanesultone (0.08 g) was added while stirring in a 1:1 molar ratio to amine groups, and the reaction was allowed to complete for 24 h at room temperature. The final product was collected and placed in a vacuum oven for 48 h.

### 2.5. Self-Assembly of Block, Random Precursors and Chemically Modified Copolymers in Aqueous Media

The main purpose of the synthesis of these specific block and statistical copolymers, as well as their derivatives, is the creation of micelles and aggregates, respectively, due to their ability to self-assemble in aqueous solutions, and the study of their physicochemical properties. Deionized water was selected as the solvent of all copolymers and the pH was adjusted to 3 with a proper amount of HCl (1 M) for the dissolution of unmodified polymers. The concentration of all solutions was regulated at 1 × 10^−3^ g/mL. Then, in this acidic environment, the study of the influence of the ionic strength was performed, adding 9 different portions of NaCl (1 M). Afterward, the pH was adjusted to 7 and 10 using the proper amount of NaOH (1 M) each time to study the response of the polymeric systems to temperature. Concerning dynamic light scattering studies, all solutions were filtered through hydrophilic PVDF 0.45 µm disposable filters before measurements. Additionally, studies were conducted through fluorescence spectroscopy, using pyrene as the tracer, to determine the CMC and CAC, assuring the formation of micelles and aggregates, respectively. For each polymeric system, eleven solutions were prepared with increasing concentration (from 10^−8^ to 10^−3^ g/mL), and pyrene was added at a ratio 1 μL pyrene solution/1 mL copolymer solution.

## 3. Results

### 3.1. Synthesis and Molecular Characterization of Block and Random Copolymers

**Scheme 1:**
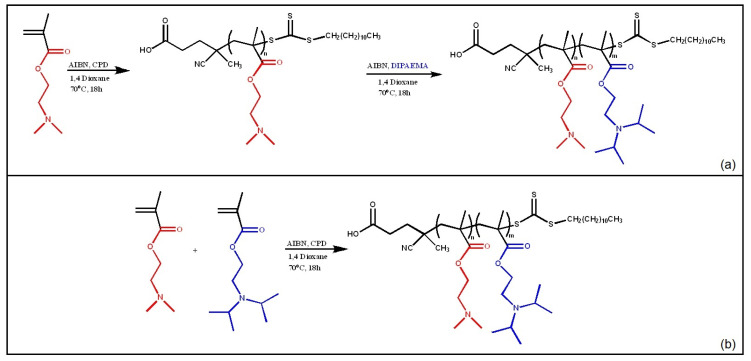
Synthetic route for obtaining (**a**) PDMAEMA-b-PDIPAEMA and (**b**) P(DMAEMA-co-DIPAEMA) copolymers via RAFT polymerization.

**Figure 1:**
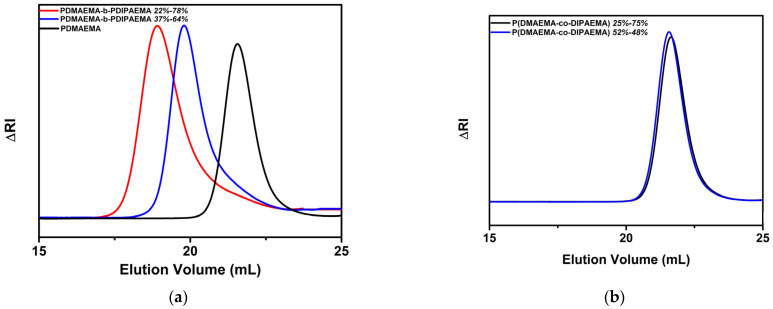
SEC chromatograms of (**a**) PDMAEMA-b-PDIPAEMA block copolymer with different compositions (red and blue curves) compared to the PDMAEMA homopolymer (black curve) and (**b**) P(DMAEMA-co-DIPAEMA) statistical copolymers with different compositions (black and blue curves).

The synthesis of two PDMAEMA-b-PDIPAEMA block copolymers and two statistical P(DMAEMA-co-DIPAEMA) copolymers was conducted via RAFT polymerization, as shown in Scheme 1, in which different colors indicate different monomeric units. The choice of CDP as the chain transfer agent was based on previous work and results from the literature [20], which have shown that CDP is an efficient CTA for the polymerization of the chosen monomers. The two diblock copolymers and the two statistical copolymers have different monomer compositions by weight. The first characterization method utilized was size exclusion chromatography (SEC), which showed that the polymerization process was successfully accomplished in each case. Two representative chromatograms are shown in Figure 1, one for the statistical copolymers and one for the diblock copolymers compared to the initial PDMAEMA homopolymer chromatogram. As observed, all peaks are unimodal, proving the controlled polymerization, and as a result, molecular mass distribution values are low, consistent with the theoretical background of RAFT polymerization.

**Figure 2:**
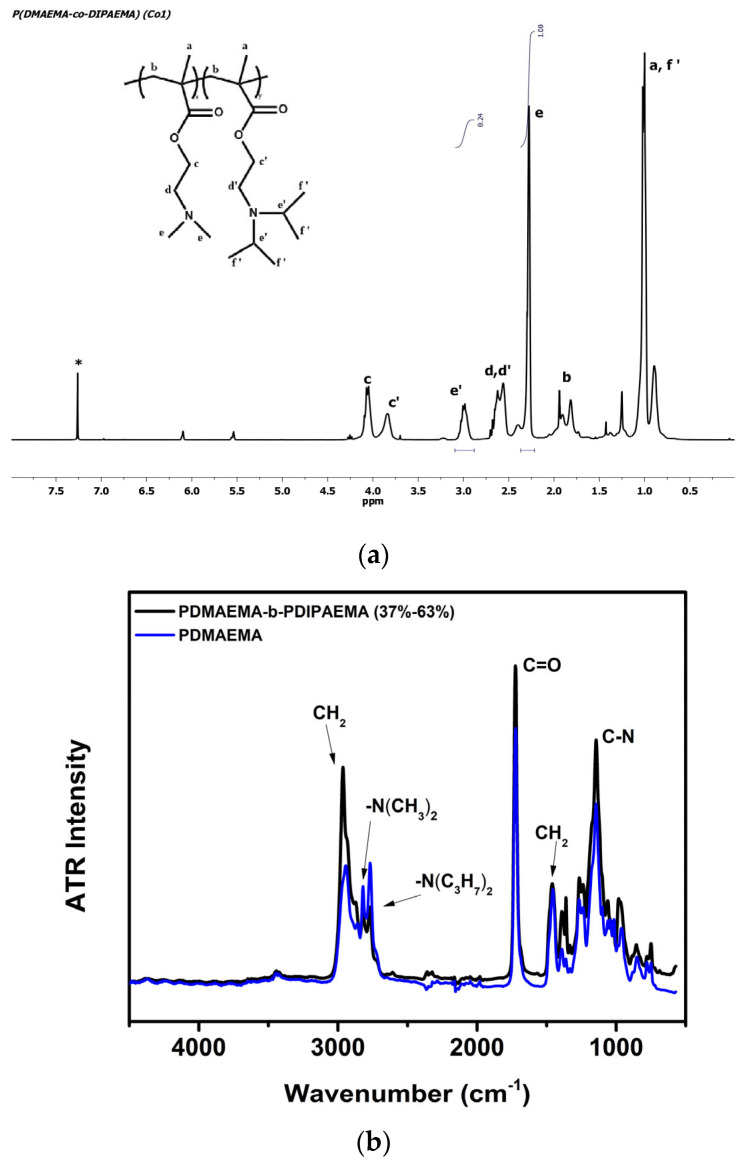
(**a**) ^1^H-NMR spectra of P(DMAEMA−co−DIPAEMA) (Co1). Letters in the spectrum correspond to the protons shown in the chemical structure on the upper left part of the figure. (**b**) ATR-FTIR spectra of diblock copolymer compared to the homopolymer.

^1^H-NMR (Figure 2a) and ATR-FTIR (Figure 2b) experiments were conducted for the identification of the chemical structure and composition of the two diblock copolymers and the two random copolymers. The first technique confirmed both qualitatively and quantitatively that both monomers are included in all copolymers, while the second one provided qualitative results. Concerning the ^1^H-NMR spectra, the quantitative analysis was achieved using the most characteristic peaks for each monomer. More specifically, for PDMAEMA the hydrogens (–CH_3_) of the two methyl groups connected to the amino group, corresponding to peak e in Figure 2a (2.32 ppm), were chosen [21]. Additionally, for PDIPAEMA, the peak appearing at 3.00 ppm was selected (peak e’ in Figure 2a), which corresponds to –CH– of the isopropyl group connected to the amino group [22,23]. These characteristic spectral peaks were used to calculate the composition of all copolymers, as presented in the Supplementary Materials. The molecular characteristics of all copolymers obtained by SEC and ^1^H-NMR are presented in Table 1.

ATR-FTIR spectroscopy was also used for the identification of the copolymers’ chemical structures. Figure 2b shows the FTIR spectra of diblock copolymer PDMAEMA-b-PDIPAEMA (B1) and PDMAEMA homopolymer. The differences in intensity between the two spectra are due to the enhancement of some vibrations, because of the presence of the second DIPAEMA block. As illustrated in Figure 2b, the peak at 2967 cm^−1^ corresponds to C–H (–CH_2_ group) stretching vibrations from both DMAEMA and DIPAEMA [17,23]. The absorption peaks at 2819 cm^−1^ and 2759 cm^−1^ are attributed to stretching vibrations of N–C of tertiary amino groups (–N(CH_3_)_2_ of DMAEMA and –N(C_3_H_7_)_2_ of DIPAEMA) of both components [17,23]. The peak appearing at 1724 cm^−1^ corresponds to C=O stretching, and the one at 1466 cm^−1^ is due to C–H (–CH_2_ group) bending vibration [17,23]. The last peak at 1140 cm^−1^ is attributed to stretching vibration of C–N bonds, which are present in both DMAEMA and DIPAEMA [17,23]. It is observed that there is an enhancement in the intensity of certain peaks, indicating the presence of the second block of DIPAEMA. The spectra obtained via FT-IR spectroscopy certified the expected chemical structure of the synthesized copolymers.

### 3.2. Synthesis and Molecular Characterization of Chemically Modified Copolymers

**Scheme 2:**
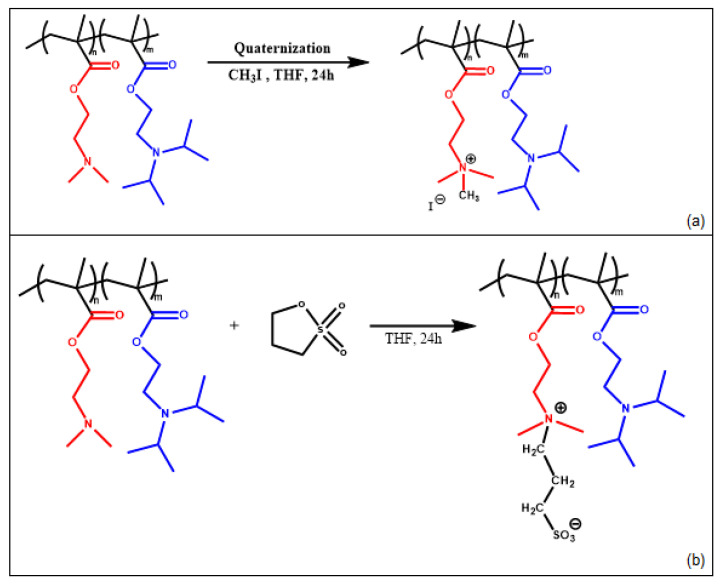
(**a**) Quaternization reaction and (**b**) sulfobetainization reaction.

The hydrophilic block of the synthesized PDMAEMA-b-PDIPAEMA and P(DMAEMA-co-DIPAEMA) copolymers was transformed to quaternary ammonium salt. Consequently, the PDMAEMA block was converted from a weak to a strong cationic polyelectrolyte. The quaternization reaction using CH_3_I and the formation of the polyelectrolyte is demonstrated in Scheme 2a, in which different colors indicate different monomeric unit. Furthermore, the amine group of PDMAEMA of the block and random copolymers was converted to a sulfobetaine group using 1,3-propanesultone, and as a consequence, the PDMAEMA block was converted to a polyzwitterion. The reaction is shown in Scheme 2b. The chemically modified copolymers were studied only with ^1^H-NMR and ATR-FTIR spectroscopies, since they were insoluble in THF due to the presence of positive and negative charges; thus, measurements with SEC chromatography could not be performed.

**Figure 3:**
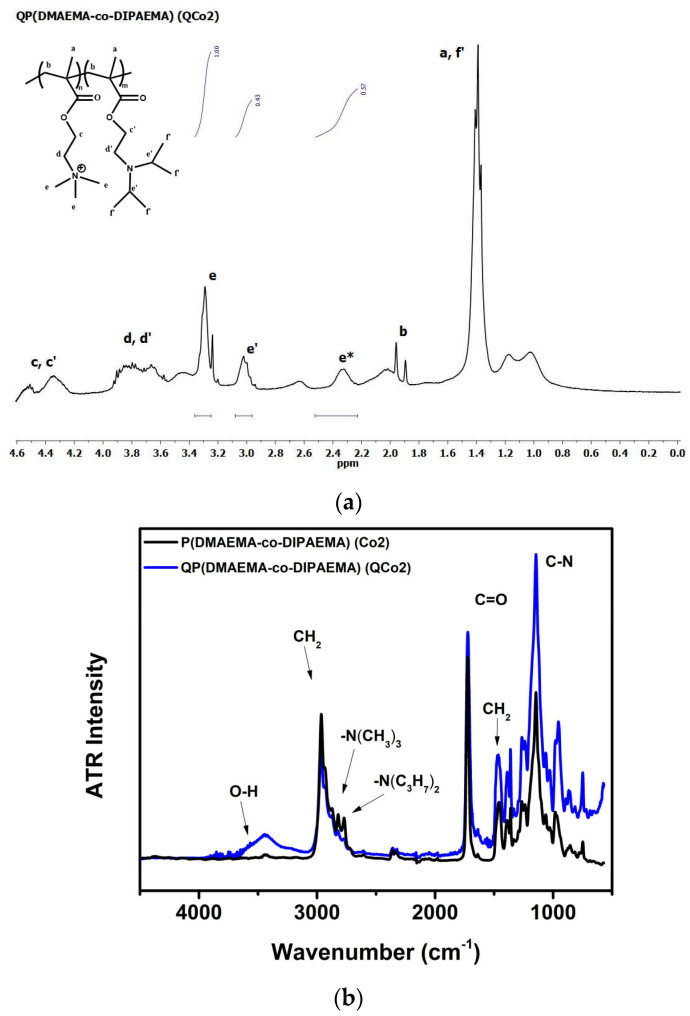
(**a**) ^1^H-NMR spectra of QP(DMAEMA−co−DIPAEMA) (QCo2). Letters in the spectrum correspond to the protons shown in the chemical structure on the upper left part of the figure. (**b**) ATR-FTIR spectra of random copolymer before (black line) and after (blue line) the quaternization.

**Figure 4:**
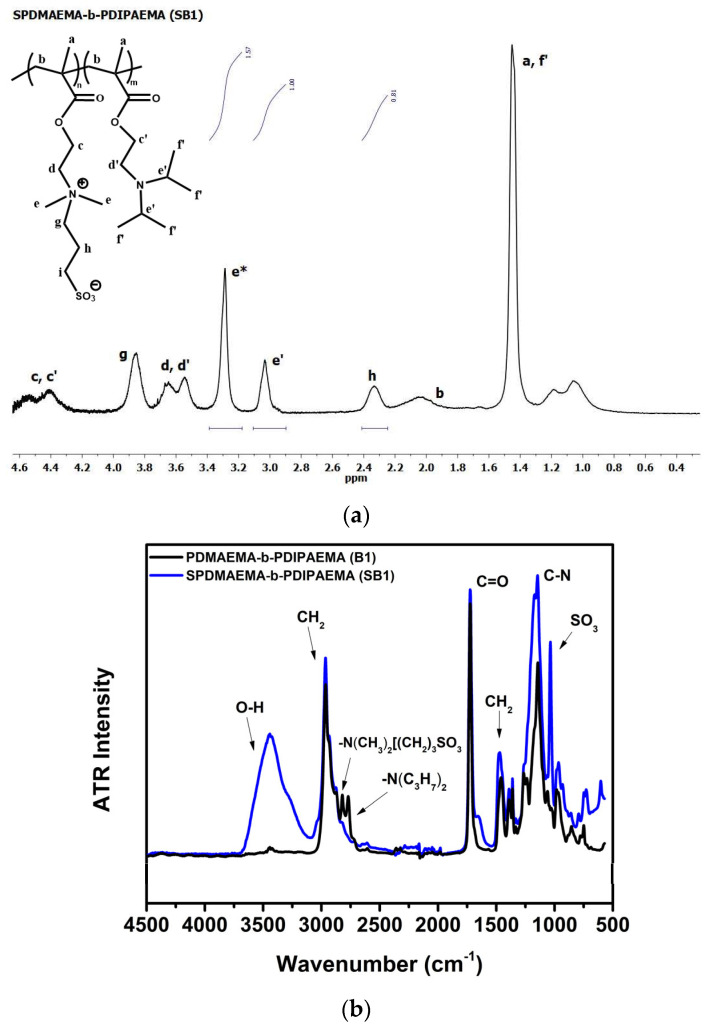
(**a**) ^1^H-NMR spectra of SPDMAEMA−b−PDIPAEMA (SB1). Letters in the spectrum correspond to the protons shown in the chemical structure on the upper left part of the figure. (**b**) ATR-FTIR spectra of block copolymer before (black line) and after (blue line) the sulfobetainization reaction.

^1^H-NMR (Figure 3a and Figure 4a) and ATR-FTIR (Figure 3b and Figure 4b) experiments were conducted for the quantitative and qualitative determination of the chemical structure and composition of the quaternized and sulfobetainized copolymers. Regarding the QPDMAEMA, the –CH_3_– hydrogens (methyl group) were chosen (peak e of Figure 3a), appearing at 3.14 ppm [24]. For the PDIPAEMA, the peak that appears at 2.88 ppm corresponds to the –CH– of the isopropyl group connected to the amino group (peak e’ of Figure 3a) [24]. Concerning the sulfobetainized copolymers, for the SPDMAEMA, the chosen hydrogens were those of –CH_2_– (methylene) at 2.19 ppm (peak h of Figure 4a), while for the PDIPAEMA, hydrogens of the isopropyl group –CH– were used (peak e’ of Figure 4a) [25]. The above-mentioned peaks were used to calculate the composition of each chemically modified copolymer. The observed shift in all peaks compared to the spectra of the unmodified copolymers is due to the different solvent that was utilized (D_2_O). The molecular characteristics of all chemically modified copolymers obtained via ^1^H-NMR are shown in Table 2. Theoretical compositions were determined assuming that the quaternization reactions and sulfobetainization reactions were 100% efficient. As indicated from the experimentally determined composition, there are DMAEMA segments which have not been converted to the preferable polyelectrolyte as a result of steric hindrance.

Additionally, regarding the ATR-FTIR spectra of the chemically modified copolymers, except the peaks that are already discussed in Section 3.1, it is obvious that there is a new peak (at ca. 3440–3450 cm^−1^) for all quaternized and sulfobetainized polymers, as shown in Figure 3b and in Figure 4b. This happens because of the intrinsic humidity of the polymers due to their increased hydrophilicity. Also, the intensity of the peak corresponding to C-N bond has increased in both cases, while in sulfobetainized copolymers, there is a second new peak corresponding to SO_3_^−^ groups [26,27,28].

### 3.3. Determination of CMC/CAC of Block and Random Copolymers

**Figure 5:**
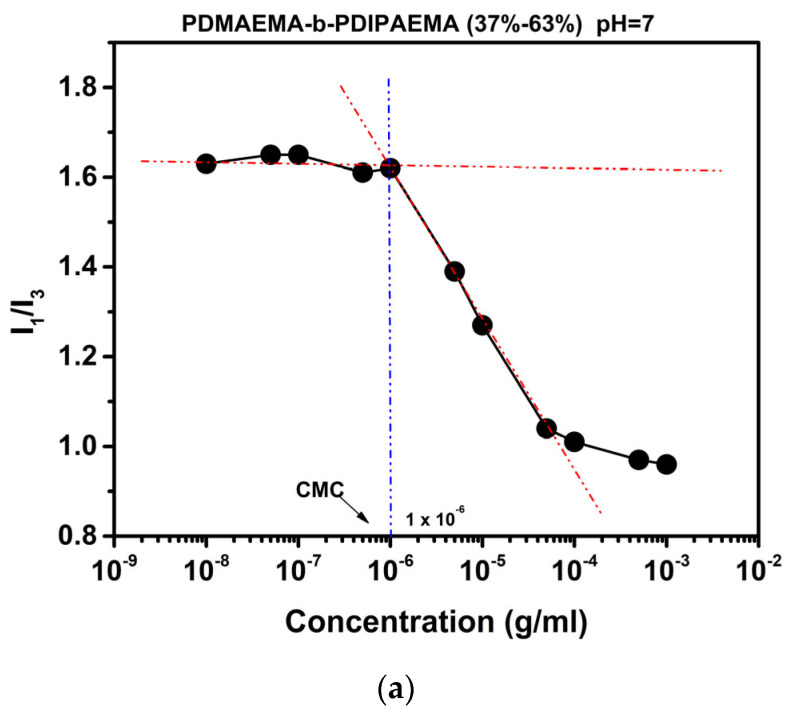

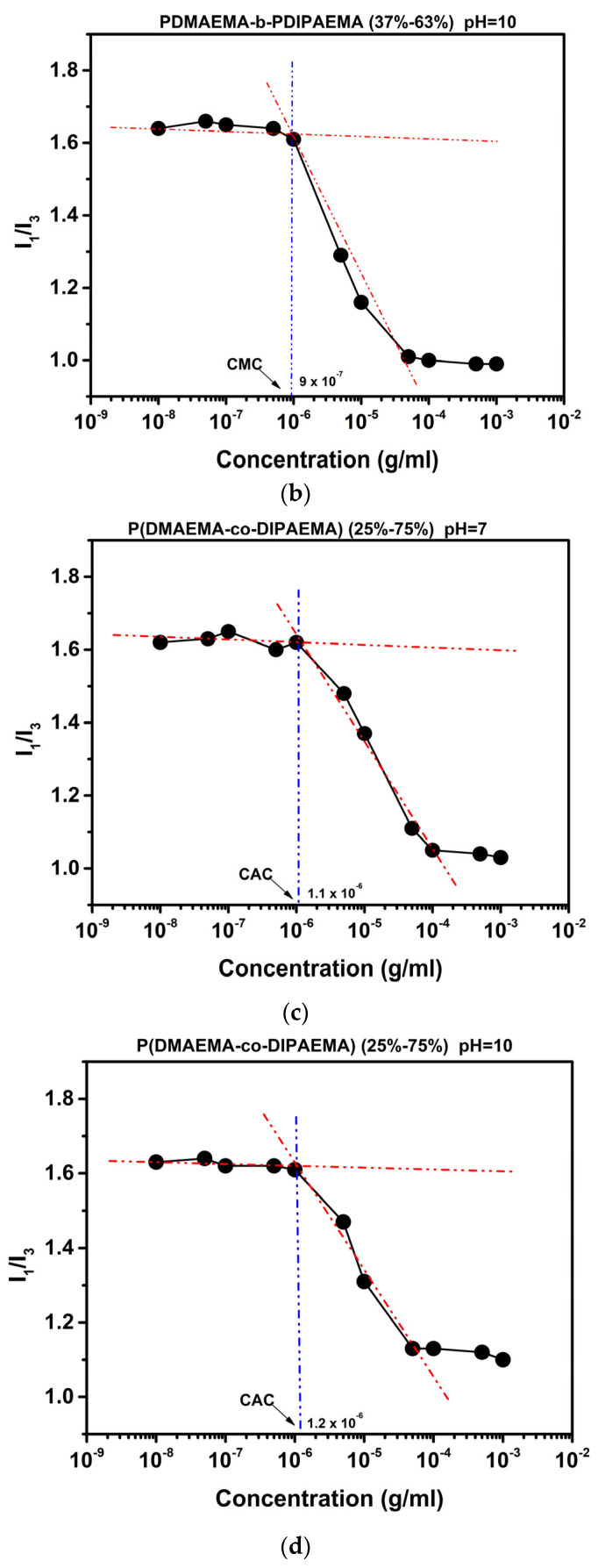
Plots of intensity ratio I_1_/I_3_ of pyrene as a function of concentration for (**a**) PDMAEMA−b−PDIPAEMA (B1) at pH = 7, (**b**) PDMAEMA−b−PDIPAEMA (B1) at pH = 10, (**c**) P(DMAEMA−co−DIPAEMA) (Co2) at pH = 7 and (**d**) P(DMAEMA−co−DIPAEMA) (Co2) at pH = 10.

The critical micellization concentration (CMC) for diblock copolymers or critical aggregation concentration (CAC) for statistical copolymers were determined for all copolymers via fluorescence spectroscopy experiments in aqueous solutions of diblock and random copolymers, using pyrene as the tracer. The fluorescence of pyrene is indicated by the first peak in a fluorescence spectrum (I_1_), and when the pyrene is trapped in the hydrophobic core of the formed nanostructure, the intensity of I_1_ decreases. CMC and CAC are defined as the concentrations corresponding to the point at which the ratio of the relative intensities of the first and third peaks, appearing in the pyrene spectrum as I_1_/I_3_, starts to decrease. Indicatively, Figure 5 shows representative plots for one diblock and one random copolymer at two different pH values (7, 10). There are three regions, the first is the plateau at low concentrations where there are no micelles or aggregates, the second is the transition region to intermediate concentrations where single chains and aggregates coexist and the third is the plateau at high concentrations where aggregates dominate in solution. CMC and CAC are determined from the first inflection point of the imaginary lines, as shown in Figure 5. The CMC and CAC values of all copolymers are given in Table 3.

The differences between the values at the two pHs for each copolymer are negligible. However, polymer Co1 is an exception since there is no value of CAC at pH = 7, at which a constant plateau value (I_1_/I_3_ ≅ 1.6) was observed for all concentrations. This value indicates the hydrophilicity of this copolymer, yet there is formation of aggregates, which is verified from DLS experiments.

### 3.4. pH Responsiveness of Block and Random Copolymers

The way pH affects aqueous solutions of all copolymers was studied with electrophoretic light scattering (ELS) at three different pH values (3, 7, 10). According to the results presented in Table 4, it can be observed that in an acidic environment, the zeta potential is positive, and as pH increases, the value of zeta potential decreases. The positive charge depicts the protonation of amine groups in an acidic environment. On the other hand, the negative charge denotes their deprotonation, which occurs when the pH of the solution is higher than 7.3 (pK_a(DMAEMA)_ = 7.3, pK_a(DIPAEMA)_ = 6.5) and is also due to the presence of deprotonated carboxyl groups present at the chain end coming from the CTA fragment [20,22]. When in a basic environment, there are a lot of hydroxide ions (OH^−^) in the solution which may absorb on the polymeric chain, conferring negative charges to the system. Additionally, it is observed that different samples have different values of zeta potentials, and this is because all copolymers exhibit a variety of architectures, compositions and interactions in the solution leading to different behaviors with changes in the solution pH.

### 3.5. Thermo-Responsiveness of Block and Random Copolymers

Aqueous solutions of polymers were studied via dynamic light scattering (DLS) to determine the effect of temperature on the formed nanostructures. PDMAEMA exhibits low critical solution temperature (LCST) in water at 40–50 °C [29]. However, LCST is different for every polymer system and depends on many factors, such as the composition, end groups and the molecular weight.

**Figure 6:**
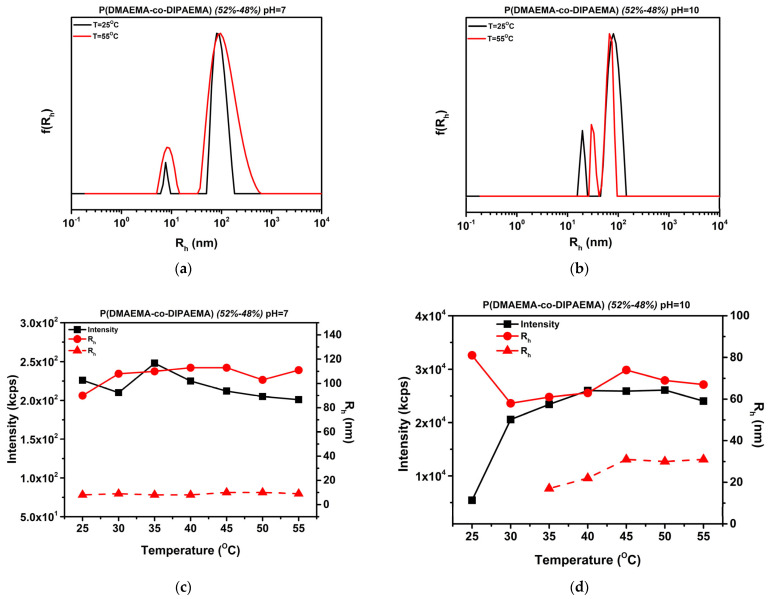
(**a**) Size distribution for random copolymer Co1 at pH = 7, (**b**) size distribution of Co1 at pH = 10, (**c**) plot of scattered light intensity and R_h_ of Co1 at pH = 7 and (**d**) plot of scattered light intensity and R_h_ of Co1 at pH = 10.

As presented in Figure 6, increasing temperature has an insignificant impact on the populations of copolymer system Co1 at pH = 7, and there is no variation in the scattered light intensity. In contrast, at pH = 10, R_h_ values of resolved peaks shift to slightly larger and slightly lower values, while the intensity increases suddenly and then remains relatively constant. Concerning the random copolymer Co2, as temperature increases, at neutral pH, there is a new population at lower R_h_ value due to the possible breaking of the larger aggregates, whereas at pH = 10, there are negligible differences in both the number of populations and the intensity of scattered light, as shown in Figure S1. Regarding the block copolymers, populations of system B1 at pH = 7 and the intensity of scattered light are not influenced by the rise in temperature, whilst at pH = 10, there is a new population with larger R_h_ due to the possible formation of micellar aggregates. In parallel, the intensity increases and then suddenly decreases, as presented in Figure S2 [19,20,22]. Lastly, for the block copolymer B2 at pH = 7, the two populations observed are combined because of PDMAEMA’s shrinkage to the center of the micelle core accompanied with decreasing scattered light intensity. At basic pH, the impacts of temperature on the aggregate populations and the intensity are rather negligible, as shown in Figure S3 [19,20,22]. The measured values of R_h_ and the intensities of all copolymer solutions at 25 °C and 55 °C are shown in Table 5. It should be noted that the values of R_h_ refer to the larger population of every copolymer, since the smaller population could be single chains or aggregates with only a small number of chains. In particular, statistical copolymers may have various kinds of aggregates due to the randomness of the chains.

### 3.6. Effect of Solution Ionic Strength

Another parameter that affects the self-assembly of aqueous charged polymer solutions is the increase in ionic strength with the gradual addition of NaCl (1 M). These experiments were conducted using DLS and at acidic pH due to the protonation of both PDMAEMA and PDIPAEMA.

**Figure 7:**
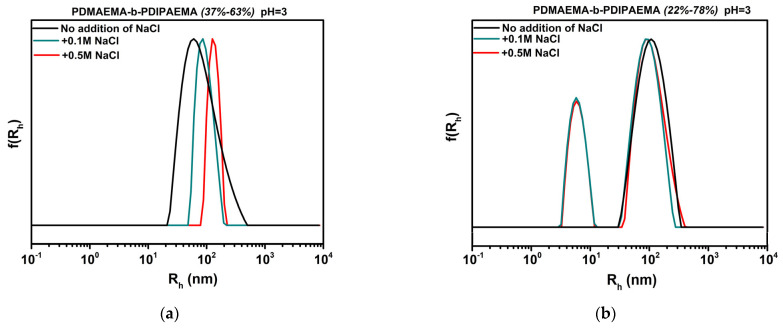

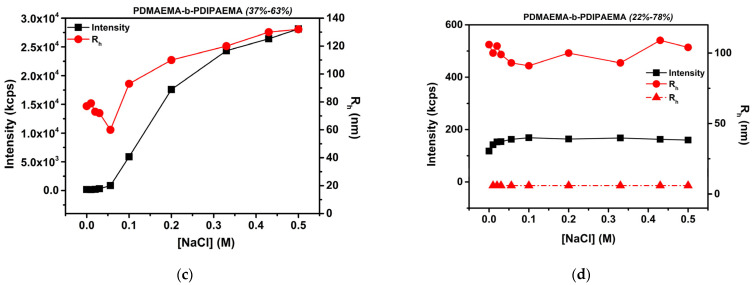
Size distributions (top) and variations in intensity and hydrodynamic radius as a function of increasing salt concentration (bottom) of PDMAEMA-b-PDIPAEMA (B1) (**a**,**c**) and PDMAEMA-b-PDIPAEMA (B2) (**b**,**d**).

As indicated in Figure 7, the addition of salt is more effective to polymer system B1 than to B2. The size distributions of B1 copolymer solution are becoming narrower and are shifted to slightly higher values of R_h_ with a simultaneous increase in scattered light intensity probably because micelles become more compact. On the other hand, copolymer B2 is rather unaffected by the presence of NaCl since it contains more PDIPAEMA, at which the amine side groups are protected by isopropyl groups [19]. Concerning statistical copolymer systems, the influence is more pronounced. More specifically and as shown in Figure S4, for Co1, both intensity and R_h_ increase with increasing salt concentration, and at 0.2 M and higher, a new population appears, probably due to the breaking of micelles. Lastly, polymeric system Co2 exhibits a sudden increase in intensity with following stabilization, while hydrodynamic radius is unaffected throughout the salt addition experiments, as indicated in Figure S5 [20]. The measured values of scattered light intensity and R_h_ of all copolymers with increasing ionic strength of the solution are presented in Table 6. It should be noted that the values of R_h_ refer to the larger population of every copolymer solution, since the smaller population could be single chains or micelles/aggregates with a small number of chains.

### 3.7. Determination of CMC/CAC of Chemically Modified Copolymers

**Figure 8:**
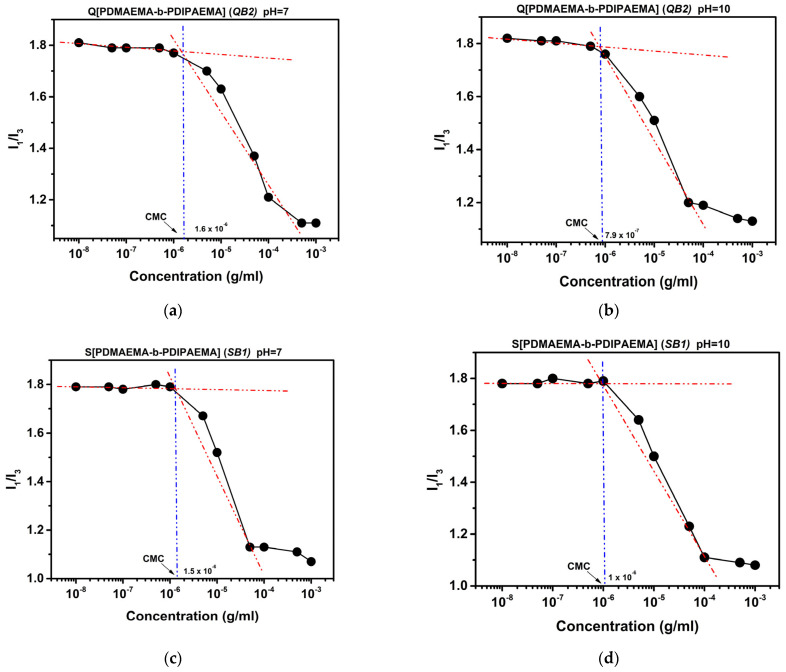
Plots of intensity ratio I_1_/I_3_ of pyrene as a function of concentration for (**a**) QPDMAEMA−b−PDIPAEMA (QB2) at pH = 7, (**b**) QPDMAEMA−b−PDIPAEMA (QB2) at pH = 10, (**c**) SPDMAEMA−b−PDIPAEMA (SB1) at pH = 7 and (**d**) SPDMAEMA−b−PDIPAEMA (SB1) at pH = 10.

The determination of CMC or CAC of the chemically modified polymers was conducted with experiments similar to the ones described in Section 3.3. Regarding the quaternized random copolymers, differences between the two pH values are insignificant, while for quaternized block polymers the differences were within one order of magnitude. This is because diblock copolymers form more well-defined structures than random copolymers, since blocks of each monomer are more distinct from each other and, therefore, are more affected by pH. On the other hand, sulfobetainized diblock and random polymers do not exhibit a general behavior, because these are polyampholytes and each one self-assembles in a different way, depending on the composition and the forces between the polymer and the solvent. Representative plots of one quaternized polymer (QB2) and one sulfobetainized polymer (SB2) at pH = 7 and pH = 10 are shown in Figure 8. The CMC and CAC values of all chemically modified polymers are given in Table 7. A general observation concerning the values of the precursor copolymers compared to the modified ones is that CMC/CAC values of most modified polymers are slightly higher, since their hydrophilicity has been increased after chemical modification.

### 3.8. pH Responsiveness of Chemically Modified Copolymers

Studies on the responsiveness of quaternized and sulfobetainized copolymers to pH changes were conducted with electrophoretic light scattering (ELS). According to the results shown in Table 8 for the quaternized polymers, zeta potential has the highest value in an acidic environment, and with increasing pH, the measured values decrease. The highly positive values are a result of the permanently positively charged quaternized amino groups. Due to the acidic pH, the remaining unmodified amino groups become protonated, and this results in the increased positive value of the zeta potential. On the other hand, as the pH changes to basic levels, a decrease in this value is observed, because the protonation degree decreases. Regarding the sulfobetainized polymers, it is noted that in acidic conditions, the measured zeta potential has a strongly positive value, and as the pH increases, this value decreases to the negative scale. These polymers are polyampholytes, that is, they have both positive and negative charges. Due to this fact, the copolymers seem vulnerable to pH changes, as proven by zeta potential values in Table 8.

### 3.9. Thermo-Responsiveness of Chemically Modified Copolymers

**Figure 9:**
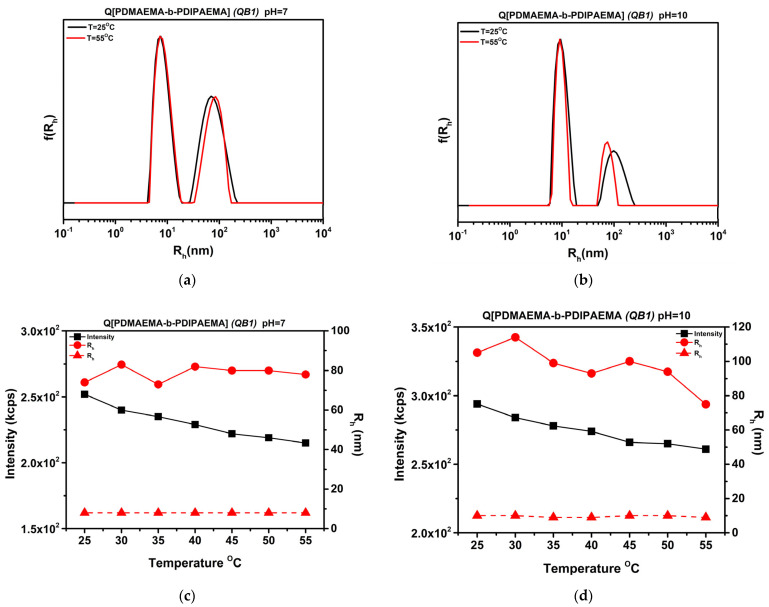
Size distributions and changes in intensity and hydrodynamic radius as a function of temperature of QPDMAEMA-b-PDIPAEMA (QB1) at two different pH values, 7 (**a**,**c**) and 10 (**b**,**d**).

The thermo-responsiveness of quaternized and sulfobetainized polymers was studied at pH = 7 and pH = 10 using dynamic light scattering (DLS). The behavior of QPDMAEMA-b-PDIPAEMA (QB1) is shown in Figure 9. Neither the intensity of scattered light nor the hydrodynamic radius at pH = 7 are affected by an increase in temperature, whereas at pH = 10, the R_h_ of the larger population slightly decreases due to the imparted hydrophilicity of the system. In addition, polymeric system QB2 remains almost unaltered at pH = 7, whilst at pH = 10, the intensity increases and the measured R_h_ is constant, indicating that existing micelles become more compact, as presented in Figure S6. Concerning the quaternized random copolymers, QCo1 at pH = 7 exhibits a constant condition, and at pH = 10, intensity decreases almost to half and R_h_ is unaffected, as shown in Figure S7. Lastly, for the QCo2 copolymer at pH = 7, the intensity of scattered light decreases, and the hydrodynamic radius increases to a small extent, whereas at pH = 10, R_h_ remains constant, as indicated in Figure S8.

Overall, the behavior of quaternized polymers was not the expected based on previous studies [17], since in this case, the presence of the PDIPAEMA block, which is slightly thermo-responsive, affects the formation or the breaking of micelles/aggregates with increasing temperature.

**Figure 10:**
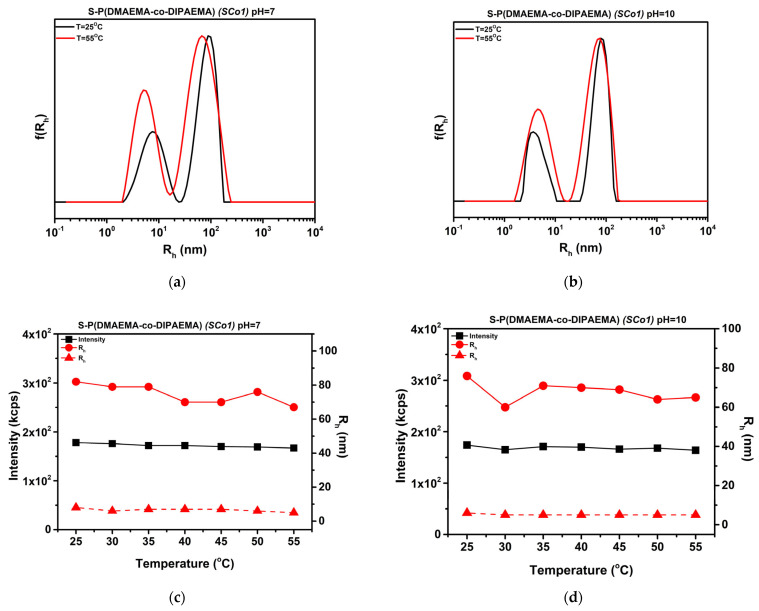
Size distributions and changes of intensity and hydrodynamic radius as a function of temperature of SP(DMAEMA-co-DIPAEMA) (SCo1) at two different pHs, 7 (**a**,**c**) and 10 (**b**,**d**).

Concerning the sulfobetainized copolymers, they exhibit quite different behavior with increasing temperature. In both neutral and basic environments, the intensity of scattered light and the hydrodynamic radius of SCo1 are not affected by a rise in temperature, as illustrated in Figure 10. The size distribution of SCo2 at pH = 7 remains unaltered with a small shift to larger values, while intensity is almost unaffected. On the other hand, SCo2 at pH = 10 is completely unaffected by the increase in temperature, as shown in Figure S9. However, copolymer SB1 shows a decrease in intensity at both pH levels, whilst hydrodynamic radius is slightly shifted to larger values, as presented in Figure S10. This means that the micelles become more compact, since the system tends to disaggregate. The decreasing intensity of scattered light may be due to the UCST behavior of the system [28,30]. Lastly, regarding copolymer SB2 at pH = 7, there is a combination of the two pre-existing populations creating one with an intermediate value of R_h_, while the intensity of scattered light is stable. At pH = 10, the behavior of populations is exactly opposite with a slightly increasing intensity. This means that some micelles break with increasing temperature, forming one population with smaller R_h_ and one with a larger one, as shown in Figure S11. The measured values of R_h_ and the intensity of both quaternized and sulfobetainized copolymers at 25 °C and 55 °C are shown in Table 9. It should be noted that the values of R_h_ refer to the larger population of every copolymer, since the smaller population could be single chains or micelles/aggregates composed of a small number of chains.

### 3.10. Effect of Ionic Strength on Chemically Modified Copolymers

**Figure 11:**
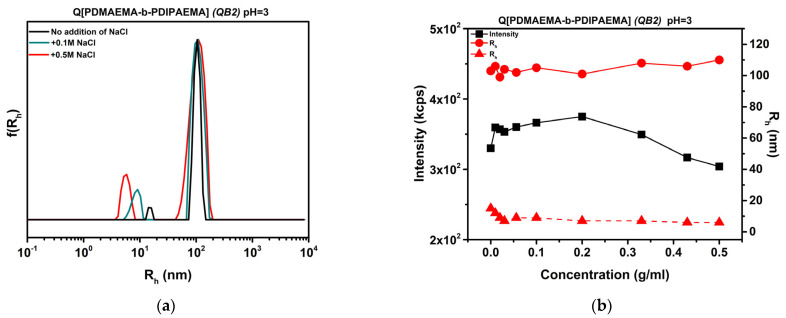
Size distributions and changes in intensity and R_h_ as a function of increasing ionic strength for copolymer QPDMAEMA-b-PDIPAEMA (QB2) aqueous solutions.

The effect of ionic strength on quaternized and sulfobetainized copolymers was studied in the same way as described in Section 3.6 using dynamic light scattering (DLS). Regarding the quaternized copolymers, all samples exhibit the same behavior with increasing salt concentration. Insignificant changes and, more specifically, decreases in scattered light intensity were observed, while hydrodynamic radius remained rather constant, as presented in Figures S12–S14 [17]. Indicatively, Figure 11 shows the size distributions and the variation in intensity and hydrodynamic radius as a function of increasing ionic strength for QPDMAEMA-b-PDIPAEMA (QB2) copolymer solutions.

**Figure 12:**
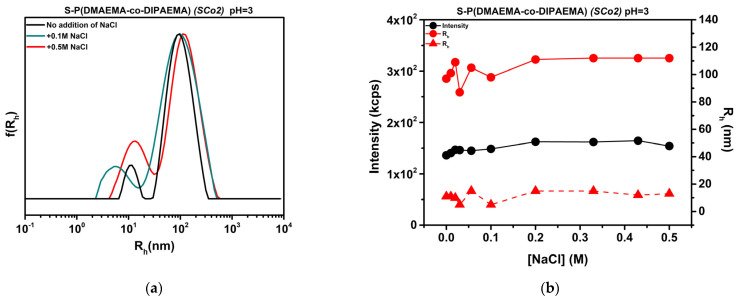
Size distributions and changes in intensity and R_h_ as a function of increasing ionic strength for copolymer SP(DMAEMA−co−DIPAEMA) (SCo2) solutions.

Regarding the sulfobetainized copolymers, their behavior was different compared to that of the quaternized copolymers. Increasing salt concentration affects the SB1 polymer, as shown by a decreasing intensity while hydrodynamic radius is constant, as illustrated in Figure S15. This behavior of the system is a result of the effect of USCT due to the presence of the PDMAEMA block [28,30]. Both SB2 and SCo1 have similar behavior, which is characterized by the hydrodynamic radius remaining constant, whereas the intensity suddenly increases upon the first addition of salt and then remains constant and in the end decreases, as shown in Figures S16 and S17. This indicates that with the first addition, the micelles or aggregates become more compact and remain so until they return to their original form. Lastly, as Figure 12 depicts, system SCo2 is unaffected by the increase in salt concentration, since both hydrodynamic radius and intensity show only slight changes. The measured values of intensity and R_h_ of quaternized and sulfobetainized copolymers upon increasing the ionic strength of the solution are presented in Table 10. It should be noted that the values of R_h_ refer to the larger population of every copolymer, since the smaller population could be single chains or micelles/aggregates with a small number of chains.

## 4. Conclusions

Two block and two statistical copolymers composed of dimethylamino ethyl methacrylate (DMAEMA) and 2-(diisopropylamino) ethyl methacrylate (DIPAEMA) monomers were synthesized successfully via RAFT polymerization with different monomer compositions. The copolymers were chemically transformed to the quaternized and sulfobetainized derivatives, creating polyelectrolytes and polyampholytes, respectively. The modifications were selective for the DMAEMA segments because of the lower steric hindrance exerted by the methyl groups on the nitrogen atom compared to the isopropyl groups in DIPAEMA segments. The copolymers were characterized using SEC, ^1^H-NMR and ATR-FTIR. Then, self-assembly studies through methods including FS, ELS and DLS were conducted to determine the response of the systems to changes in external factors, such as the temperature, pH and ionic strength of the solution. The precursor copolymers were sensitive to these environmental changes due to their varied composition and the different nanostructures that are formed. Regarding the chemically modified copolymers, it was observed that these systems do not exhibit a general behavior, but this depends on the copolymer composition and the electrostatic interactions, since the modified copolymers are strong polyelectrolytes and polyampholytes, respectively. Generally, all copolymers, both the precursor and the chemically modified, behave differently upon changes in external factors because of the variety of architectures, compositions and interactions in the solutions. In conclusion, the copolymers PDMAEMA-b-PDIPAEMA and P(DMAEMA-co-DIPAEMA), as well as their derivatives, exhibited interesting properties from the point of view of the self-assembled nanostructures that they form in aqueous solutions. In future studies, chemically modified copolymers, especially polyampholytes, could be utilized for drug delivery, while cationic polyelectrolytes could be used for the complexation of macromolecules, such as DNA, RNA and proteins, or other charged species.

## Figures and Tables

**Table 1:** Molecular characteristics of amphiphilic diblock and random copolymers synthesized.

Copolymer	M_w_ (×10^3^ g/mol) ^1^	M_w_/M_n_ ^1^	wt% PDMAEMA ^2^ (mole%)	wt% PDIPAEMA ^2^ (mole%)
PDMAEMA-b-PDIPAEMA (B1)	16	1.22	37 (43)	63 (57)
PDMAEMA-b-PDIPAEMA (B2)	30.5	1.21	22 (28)	78 (72)
P(DMAEMA-co-DIPAEMA) (Co1)	8.2	1.18	52 (60)	48 (40)
P(DMAEMA-co-DIPAEMA) (Co2)	7.8	1.17	25 (31)	75 (69)

^1^ Determined via SEC chromatography. ^2^ Determined via ^1^H-NMR (mole% is given in parenthesis).

**Table 2:** Molecular characteristics of all chemically modified polymers.

Copolymer	M_w_ (×10^3^ g/mol)	wt% QPDMAEMA_th_	wt% PDIPAEMA_th_	wt% QPDMAEMA_exp_ (mole%) ^1^	wt% PDIPAEMA_exp_ (mole%) ^1^	wt% PDMAEMA_exp_ (mole%) ^1^
QPDMAEMA-b-PDIPAEMA (QB1)	21.1	52	48	48 (53)	52 (47)	-
QPDMAEMA-b-PDIPAEMA (QB2)	36.4	35	65	31 (36)	69 (64)	-
QP(DMAEMA-co-DIPAEMA) (QCo1)	12	67	33	51 (54)	38 (32)	11 (13)
QP(DMAEMA-co-DIPAEMA) (QCo2)	9.5	38	62	35 (38)	49 (43)	16 (19)
SPDMAEMA-b-PDIPAEMA (SB1)	20.4	51	49	44 (36)	42 (44)	14 (20)
SPDMAEMA-b-PDIPAEMA (SB2)	35.5	33	67	27 (21)	60 (61)	13 (18)
SP(DMAEMA-co-DIPAEMA) (SCo1)	11.5	65	35	45 (37)	36 (38)	19 (25)
SP(DMAEMA-co-DIPAEMA) (SCo2)	9.2	36	64	35 (28)	51 (53)	14 (19)

^1^ Determined via ^1^H-NMR.

**Table 3:** CMC and CAC values for all copolymers.

Copolymer	CMC/CAC pH = 7 (g/mL)	CMC/CAC pH = 10 (g/mL)
PDMAEMA-b-PDIPAEMA (B1)	1 × 10^−6 (a)^	9 × 10^−7 (a)^
PDMAEMA-b-PDIPAEMA (B2)	1 × 10^−6 (a)^	1 × 10^−6 (a)^
P(DMAEMA-co-DIPAEMA) (Co1)	-	1 × 10^−6 (b)^
P(DMAEMA-co-DIPAEMA) (Co2)	1.1 × 10^−6 (b)^	1.2 × 10^−6 (b)^

^a^ Corresponds to CMC value. ^b^ Corresponds to CAC value.

**Table 4:** Zeta potential values of all copolymer solutions.

Copolymer	Zeta Potential (mV) (pH = 3)	Zeta Potential (mV) (pH = 7)	Zeta Potential (mV) (pH = 10)
PDMAEMA-b-PDIPAEMA (B1)	+42.3	+17.4	−22.8
PDMAEMA-b-PDIPAEMA (B2)	+46.6	+27.4	−8.53
P(DMAEMA-co-DIPAEMA) (Co1)	+7.25	+5.95	−10
P(DMAEMA-co-DIPAEMA) (Co2)	+68.4	+35.4	−70.4

**Table 5:** Values of scattered light intensity and hydrodynamic radius depending on pH and temperature for the two block and two random copolymers in water.

Copolymer	pH = 7				pH = 10			
	T = 25 °C		T = 55 °C		T = 25 °C		T = 55 °C	
	Int (kcps)	R_h_^cum^ (nm)	Int (kcps)	R_h_^cum^ (nm)	Int (kcps)	R_h_^cum^ (nm)	Int (kcps)	R_h_^cum^ (nm)
PDMAEMA-b-PDIPAEMA [B1]	1935	33	1903	32	2167	78	1864	75
PDMAEMA-b-PDIPAEMA [B2]	3620	89	2454	52	1122	417	827	413
P(DMAEMA-co-DIPAEMA) [Co1]	226	90	201	111	5430	81	4032	67
P(DMAEMA-co-DIPAEMA) [Co2]	470	87	568	98	32,000	103	28,000	104

**Table 6:** Scattered light intensity and hydrodynamic radius dependence on salt concentration of aqueous solution for block and random copolymers.

Copolymer	pH = 3			
	No NaCl		0.5 M NaCl	
	Int (kcps)	R_h_^cum^ (nm)	Int (kcps)	R_h_^cum^ (nm)
PDMAEMA-b-PDIPAEMA [B1]	183	77	289	132
PDMAEMA-b-PDIPAEMA [B2]	118	106	160	104
P(DMAEMA-co-DIPAEMA) [Co1]	118	89	236	119
P(DMAEMA-co-DIPAEMA) [Co2]	580	77	848	93

**Table 7:** CMC and CAC values for all chemically modified copolymers.

Copolymer	CMC/CAC pH = 7 (g/mL)	CMC/CAC pH = 10 (g/mL)
QPDMAEMA-b-PDIPAEMA (QB1)	1 × 10^−5 (a)^	2.3 × 10^−6 (a)^
QPDMAEMA-b-PDIPAEMA (QB2)	1.6 × 10^−6 (a)^	7.9 × 10^−7 (a)^
QP(DMAEMA-co-DIPAEMA) (QCo1)	3.3 × 10^−5 (b)^	7 × 10^−5 (b)^
QP(DMAEMA-co-DIPAEMA) (QCo2)	4.2 × 10^−6 (b)^	3.8 × 10^−6 (b)^
SPDMAEMA-b-PDIPAEMA (SB1)	1.5 × 10^−6 (a)^	1 × 10^−6 (a)^
SPDMAEMA-b-PDIPAEMA (SB2)	7.9 × 10^−7 (a)^	5.4 × 10^−7 (a)^
SP(DMAEMA-co-DIPAEMA) (SCo1)	1.1 × 10^−4 (b)^	1 × 10^−4 (b)^
SP(DMAEMA-co-DIPAEMA) (SCo2)	2 × 10^−5 (b)^	6.2 × 10^−6 (b)^

^a^ Corresponds to CMC value. ^b^ Corresponds to CAC value.

**Table 8:** Zeta potential values of all quaternized and sulfobetainized polymers.

Copolymer	Zeta Potential (mV) pH = 3	Zeta Potential (mV) pH = 7	Zeta Potential (mV) pH = 10
QPDMAEMA-b-PDIPAEMA (QB1)	+37.8	+27.8	+21.6
QPDMAEMA-b-PDIPAEMA (QB2)	+39.4	+35.2	+32.4
QP(DMAEMA-co-DIPAEMA) (QCo1)	+59.1	+31.4	+28.2
QP(DMAEMA-co-DIPAEMA) (QCo2)	+50.5	+31.2	+24.2
SPDMAEMA-b-PDIPAEMA (SB1)	+40.1	+0.72	−25.6
SPDMAEMA-b-PDIPAEMA (SB2)	+53.5	+37.7	−29.0
SP(DMAEMA-co-DIPAEMA) (SCo1)	+51.8	+16.5	−27.8
SP(DMAEMA-co-DIPAEMA) (SCo2)	+48.1	+29.9	−38.3

**Table 9:** Scattered light intensity and hydrodynamic radius dependence on pH and temperature for the quaternized and sulfobetainized copolymers.

Copolymer	pH = 7				pH = 10			
	T = 25 °C		T = 55 °C		T = 25 °C		T = 55 °C	
	Int (kcps)	R_h_^cum^ (nm)	Int (kcps)	R_h_^cum^ (nm)	Int (kcps)	R_h_^cum^ (nm)	Int (kcps)	R_h_^cum^ (nm)
QPDMAEMA-b-PDIPAEMA [QB1]	252	74	215	78	294	105	261	75
QPDMAEMA-b-PDIPAEMA [QB2]	618	83	630	93	669	89	806	95
QP(DMAEMA-co-DIPAEMA) [QCo1]	99	112	101	115	329	106	152	102
QP(DMAEMA-co-DIPAEMA) [QCo2]	496	61	240	90	600	82	589	85
SPDMAEMA-b-PDIPAEMA [SB1]	32,200	66	22,451	58	32,400	66	26,400	88
SPDMAEMA-b-PDIPAEMA [SB2]	85,500	90	84,800	75	54,300	70	58,800	94
SP(DMAEMA-co-DIPAEMA) [SCo1]	178	82	167	67	174	76	164	65
SP(DMAEMA-co-DIPAEMA) [SCo2]	446	290	430	238	525	68	547	82

**Table 10:** Scattered light intensity and hydrodynamic radius dependence on salt concentration for quaternized and sulfobetainized copolymers.

Copolymer	pH = 3			
	No NaCl		0.5 M NaCl	
	Int (kcps)	R_h_^cum^ (nm)	Int (kcps)	R_h_^cum^ (nm)
QPDMAEMA-b-PDIPAEMA (QB1)	328	84	250	96
QPDMAEMA-b-PDIPAEMA (QB2)	330	103	304	110
QP(DMAEMA-co-DIPAEMA) (QCo1)	262	62	200	65
QP(DMAEMA-co-DIPAEMA) (QCo2)	100	73	80	68
SPDMAEMA-b-PDIPAEMA (SB1)	808	91	570	94
SPDMAEMA-b-PDIPAEMA (SB2)	284	108	290	113
SP(DMAEMA-co-DIPAEMA) (SCo1)	367	94	232	80
SP(DMAEMA-co-DIPAEMA) (SCo2)	136	97	154	112

## Data Availability

Data are available upon request.

## Supplementary Materials

The following supporting information can be downloaded at https://www.mdpi.com/article/10.3390/polym16091284/s1. Figure S1. Size distributions and changes in intensity and hydrodynamic radius as a function of temperature for P(DMAEMA-co-DIPAEMA) (Co2) at two different pHs, 7 (a, c respectively) and 10 (b, d respectively). Figure S2. Size distributions and changes in intensity and hydrodynamic radius as a function of temperature for PDMAEMA-b-PDIPAEMA (B1) at two different pHs, 7 (a, c respectively) and 10 (b, d respectively). Figure S3. Size distributions and changes in intensity and hydrodynamic radius as a function of temperature for PDMAEMA-b-PDIPAEMA (B2) at two different pHs, 7 (a, c respectively) and 10 (b, d respectively). Figure S4. Size distributions and changes in intensity and Rh as a function of increasing ionic strength for copolymer P(DMAEMA-co-DIPAEMA) (Co1) aqueous solutions. Figure S5. Size distributions and changes in intensity and Rh as a function of increasing ionic strength for copolymer P(DMAEMA-co-DIPAEMA) (Co2) aqueous solutions. Figure S6. Size distributions and changes in intensity and hydrodynamic radius as a function of temperature for QPDMAEMA-b-PDIPAEMA (QB2) at two different pHs, 7 (a, c respectively) and 10 (b, d respectively). Figure S7. Size distributions and changes in intensity and hydrodynamic radius as a function of temperature for QP(DMAEMA-co-DIPAEMA) (QCo1) at two different pHs, 7 (a, c respectively) and 10 (b, d respectively). Figure S8. Size distributions and changes in intensity and hydrodynamic radius as a function of temperature for QP(DMAEMA-co-DIPAEMA) (QCo2) at two different pHs, 7 (a, c respectively) and 10 (b, d respectively). Figure S9. Size distributions and changes in intensity and hydrodynamic radius as a function of temperature for SP(DMAEMA-co-DIPAEMA) (SCo2) at two different pHs, 7 (a, c respectively) and 10 (b, d respectively). Figure S10. Size distributions and changes in intensity and hydrodynamic radius as a function of temperature for SPDMAEMA-b-PDIPAEMA (SB1) at two different pHs, 7 (a, c respectively) and 10 (b, d respectively). Figure S11. Size distributions and changes in intensity and hydrodynamic radius as a function of temperature for SPDMAEMA-b-PDIPAEMA (SB2) at two different pHs, 7 (a, c respectively) and 10 (b, d respectively). Figure S12. Size distributions and changes in intensity and Rh as a function of increasing ionic strength for copolymer QPDMAEMA-b-PDIPAEMA (QB1) aqueous solutions. Figure S13. Size distributions and changes in intensity and Rh as a function of increasing ionic strength for copolymer QP(DMAEMA-co-DIPAEMA) (QCo1) aqueous solutions. Figure S14. Size distributions and changes in intensity and Rh as a function of increasing ionic strength for copolymer QP(DMAEMA-co-DIPAEMA) (QCo2) aqueous solutions. Figure S15. Size distributions and changes in intensity and Rh as a function of increasing ionic strength for copolymer SPDMAEMA-b-PDIPAEMA (SB1) aqueous solutions. Figure S16. Size distributions and changes in intensity and Rh as a function of increasing ionic strength for copolymer SPDMAEMA-b-PDIPAEMA (SB2) aqueous solutions. Figure S17. Size distributions and changes in intensity and Rh as a function of increasing ionic strength for copolymer SP(DMAEMA-co-DIPAEMA) (SCo1) aqueous solutions.
