# Supplementary material for: Block and Statistical Copolymers of Methacrylate Monomers with Dimethylamino and Diisopropylamino Groups on the Side Chains: Synthesis, Chemical Modification and Self-Assembly in Aqueous Media
Source: Polymers (Basel). 2024 May 3;16(9):1284. doi: 10.3390/polym16091284 (PMC11085793; doi:10.3390/polym16091284)
Supplement: Supplementary file 1 [file polymers-16-01284-s001.zip › polymers-2961269-supplementary.pdf]

Supplementary Materials

Block and statistical copolymers of methacrylate monomers with dimethylamino and diisopropylamino groups on the side chains: Synthesis, chemical modification and self- assembly in aqueous media.

Kalliopi Makri [1] and Stergios Pispas [1,*]

[1] Theoretical and Physical Chemistry Institute, National Hellenic Research Foundation, 48 Vassileos Constantinou Avenue, 11635 Athens Greece
* Correspondence: pispas@eie.gr

Calculation of copolymer composition

The most characteristic peaks of each monomer were used to calculate the composition of the synthesized copolymers. More specifically, for PDMAEMA the hydrogens (-CH_3) of the two methyls connected to the amino-group, corresponding to peak e in Figure 2(a) (2.32ppm), were chosen and for PDIPAEMA, the peak appearing at 3.00ppm was selected (peak e' in Figure 2(a)), which corresponds to -CH- of the isopropyl-group connected to the amino-group. Then, the area of each peak was divided with the number of hydrogens corresponding to the peak and the result was multiplied by the molecular weight of the corresponding monomeric unit. Dividing the final result with the sum of the peaks, the weight percent of each monomeric unit was determined.

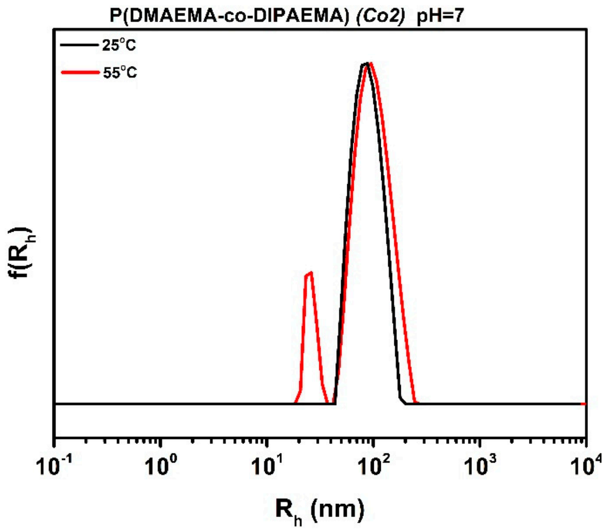

(a)

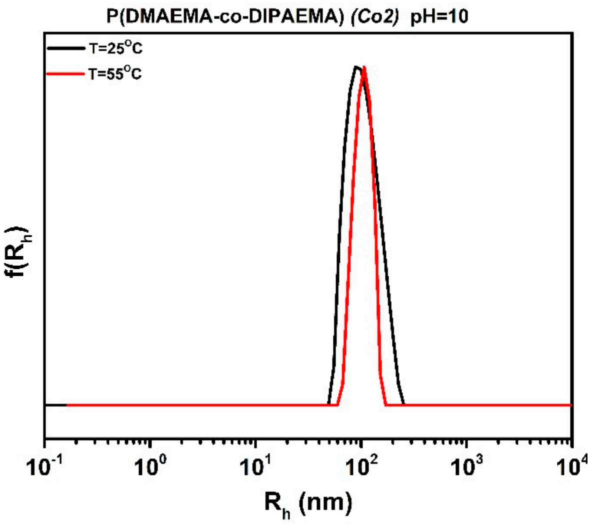

(b)

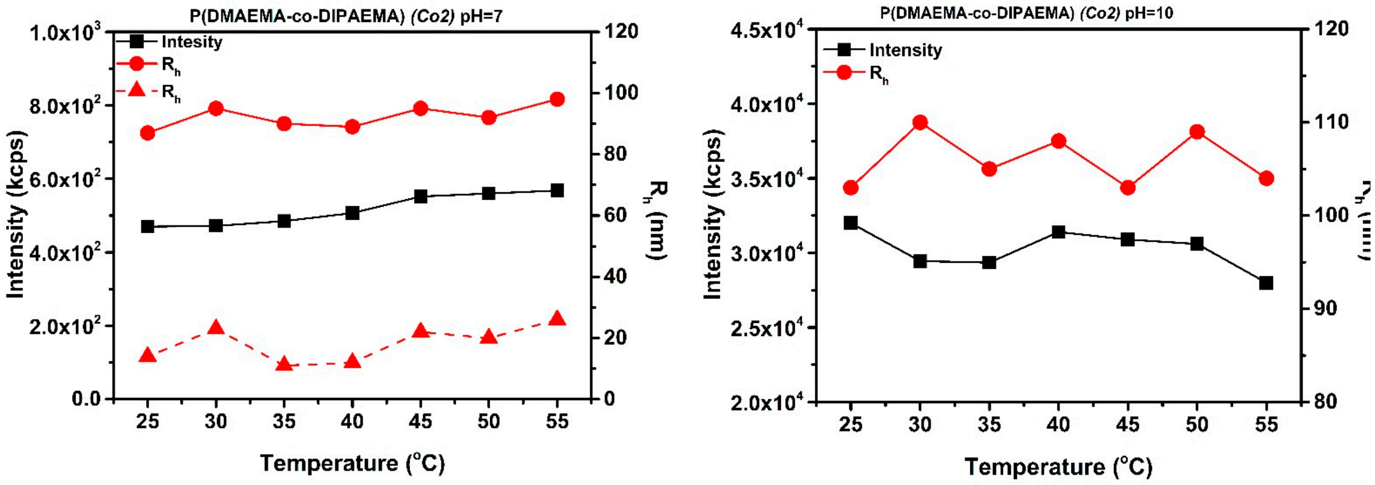

(c) (d)

Figure S1. Size distributions and changes of intensity and hydrodynamic radius as a function of temperature for P(DMAEMA-co-DIPAEMA) (Co2) at two different pHs, 7 (**a**, **c** respectively) and 10 (**b**, **d** respectively).

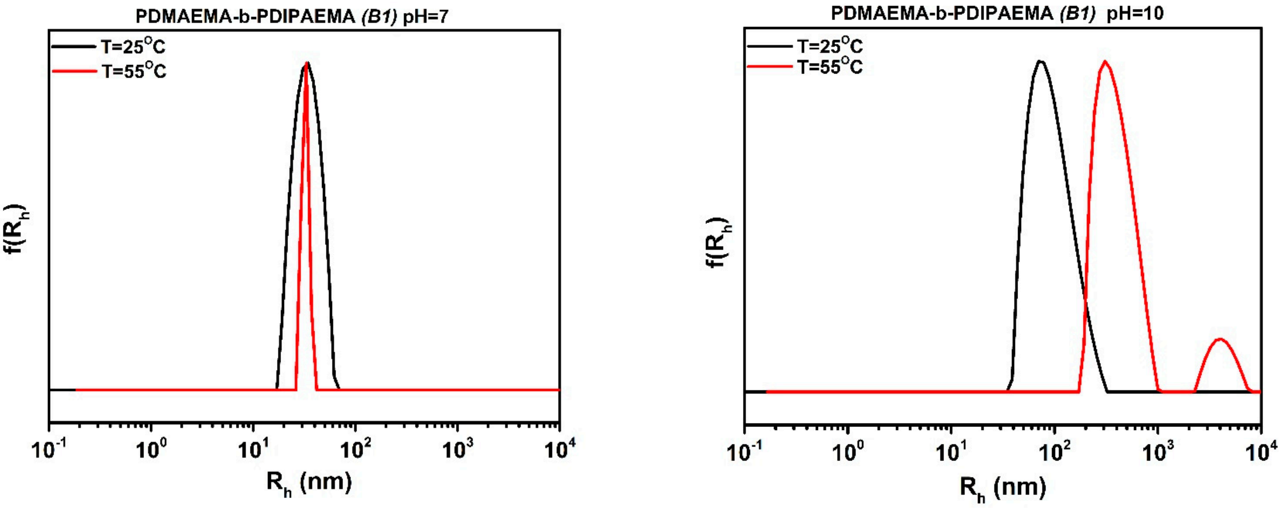

(a) (b)

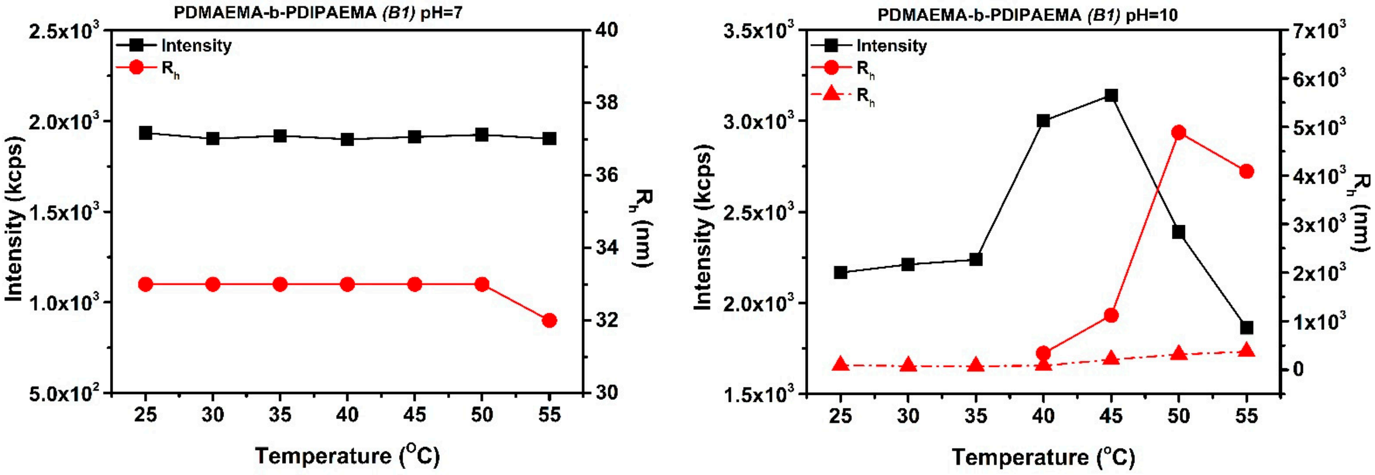

(c) (d)

Figure S2. Size distributions and changes of intensity and hydrodynamic radius as a function of temperature for PDMAEMA-b-PDIPAEMA (B1) at two different pHs, 7 (**a**, **c** respectively) and 10 (**b**, **d** respectively).

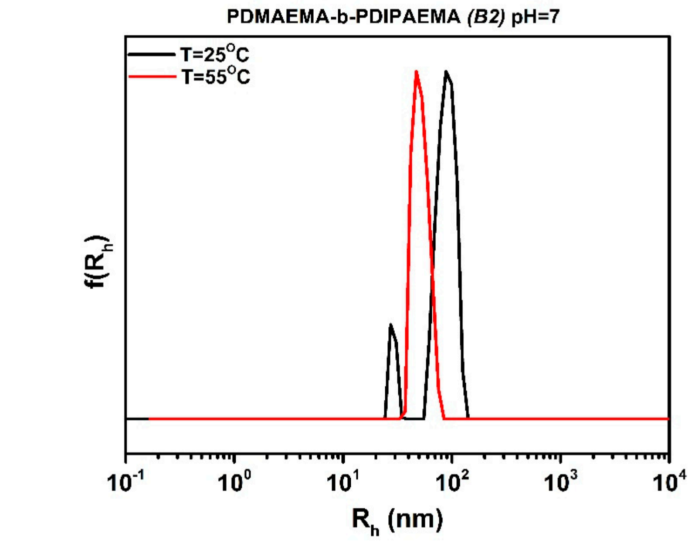

(a)

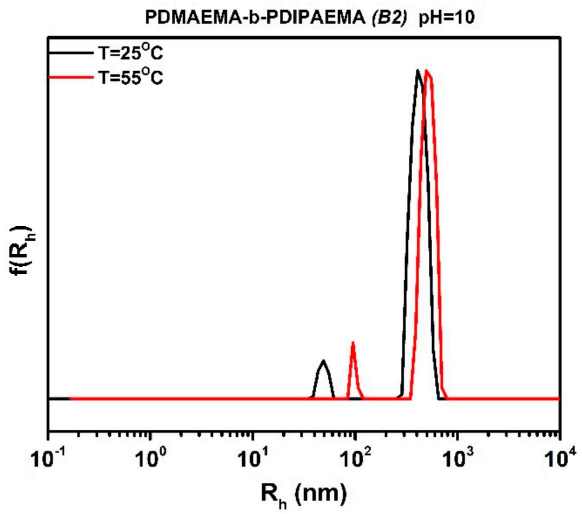

(b)

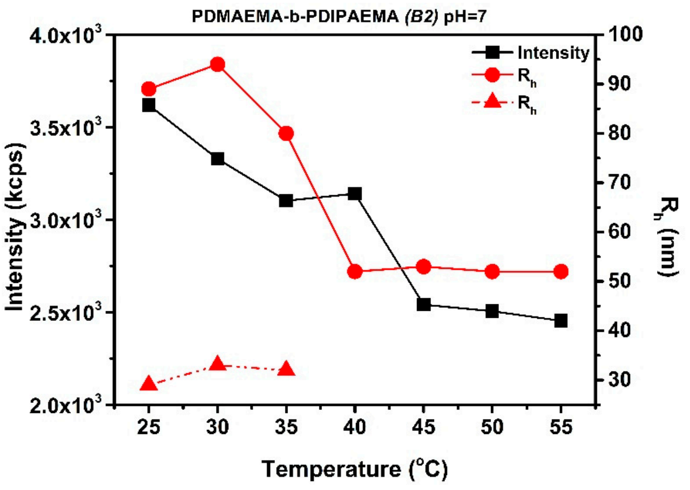

(c)

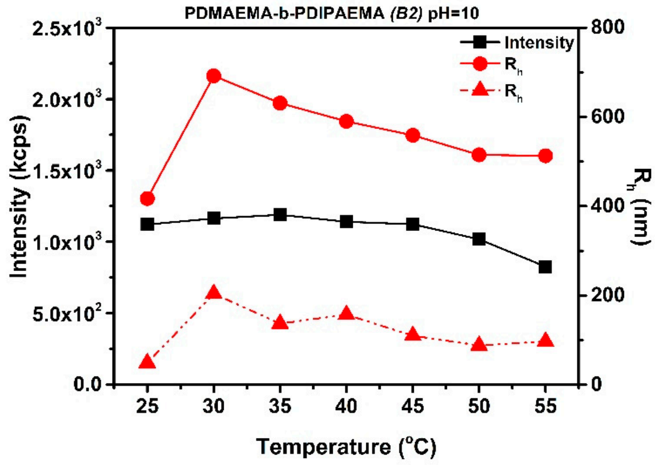

(d)

Figure S3. Size distributions and changes of intensity and hydrodynamic radius as a function of temperature for PDMAEMA-b-PDIPAEMA (B2) at two different pHs, 7 (**a**, **c** respectively) and 10 (**b**, **d** respectively).

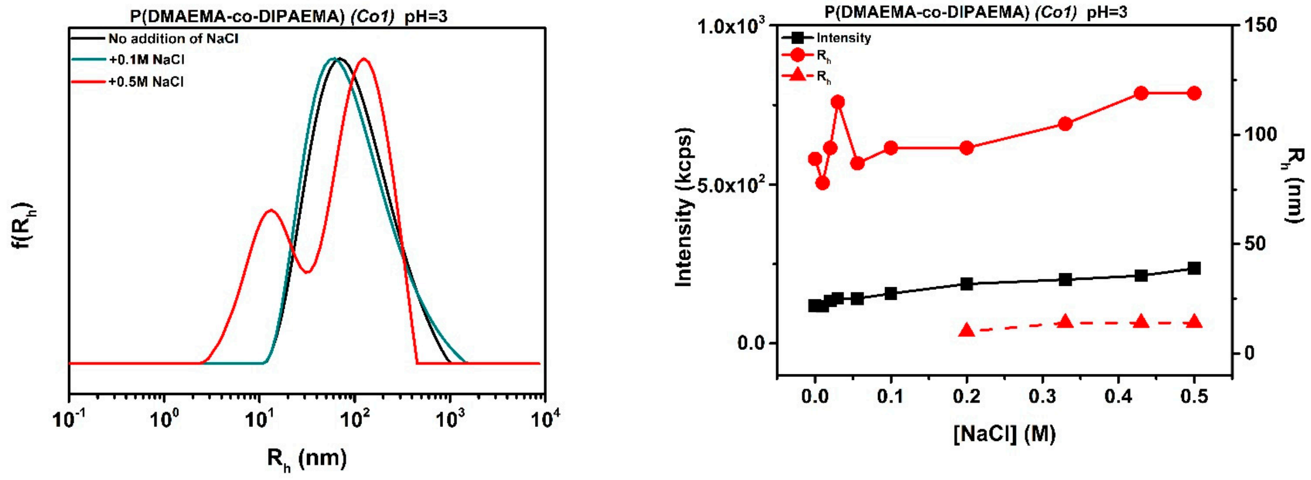

(a) (b)

Figure S4. Size distributions and changes of intensity and R_h as a function of increasing ionic strength for copolymer P(DMAEMA-co-DIPAEMA) (Co1) aqueous solutions.

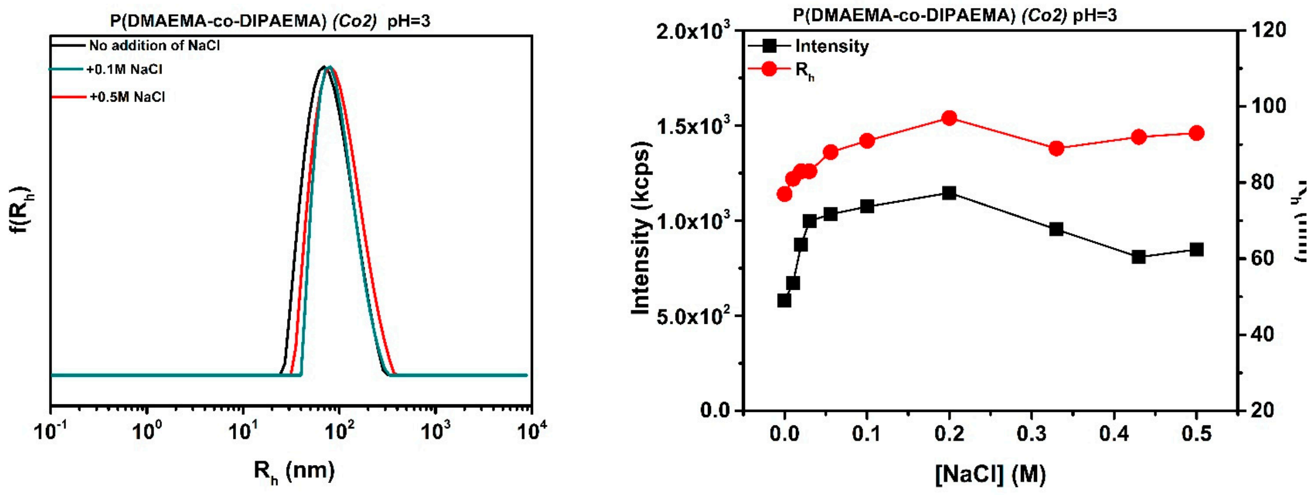

(c) (d)

Figure S5. Size distributions and changes of intensity and R_h as a function of increasing ionic strength for copolymer P(DMAEMA-co-DIPAEMA) (Co2) aqueous solutions.

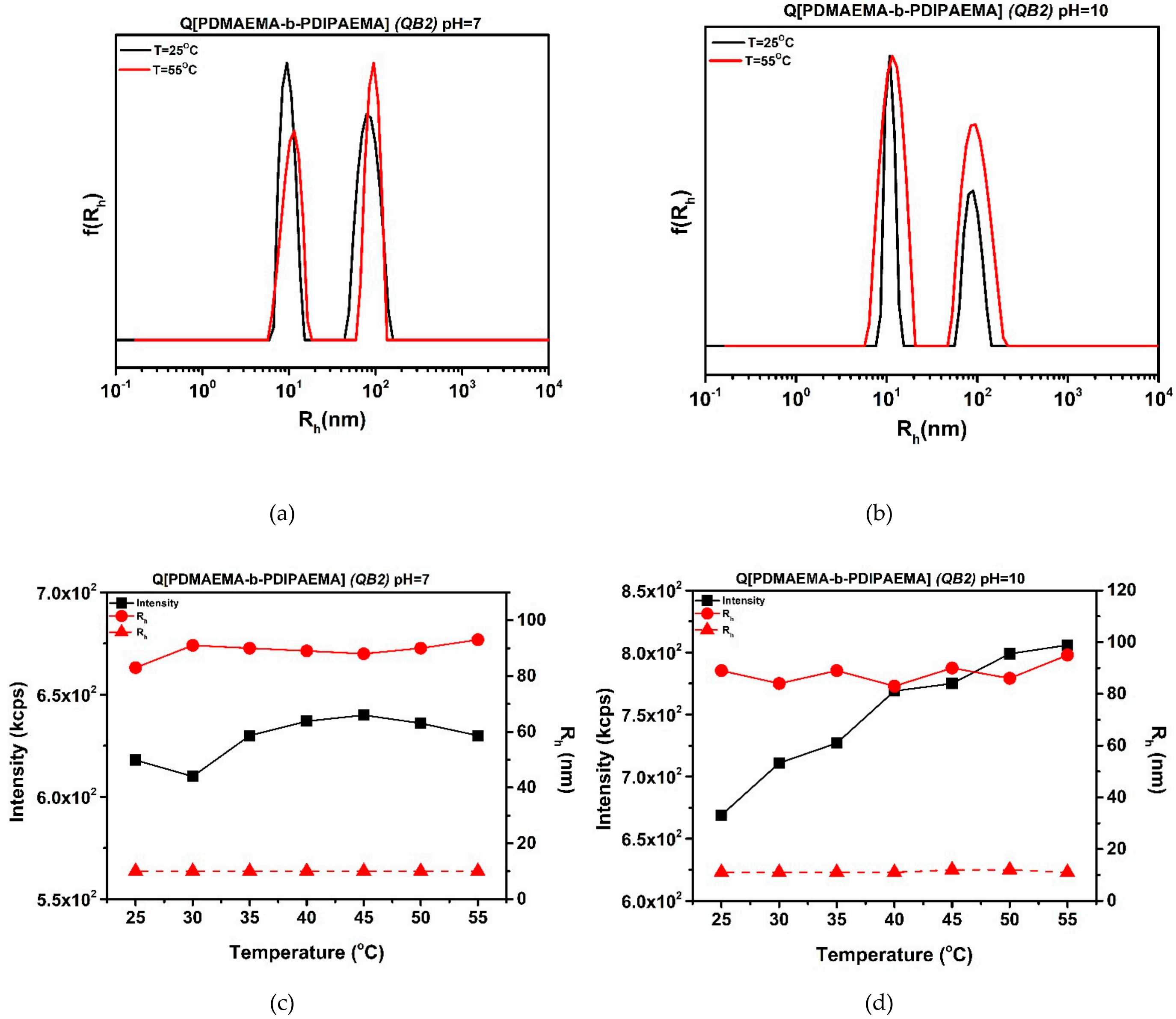

Figure S6. Size distributions and changes of intensity and hydrodynamic radius as a function of temperature for QPDMAEMA-b-PDIPAEMA (QB2) at two different pHs, 7 (**a**, **c** respectively) and 10 (**b**, **d** respectively).

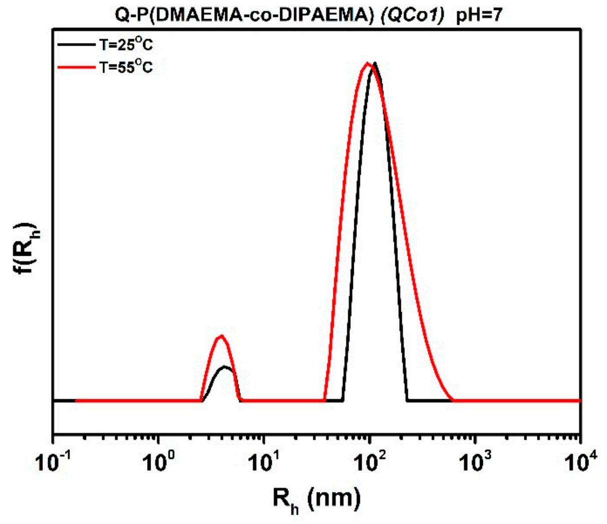

(a)

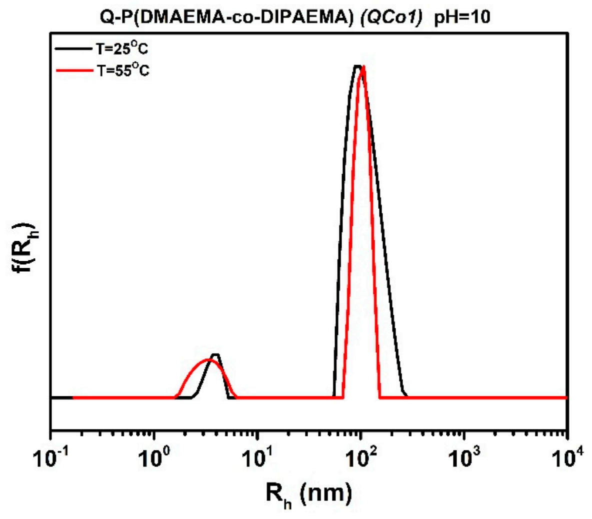

(b)

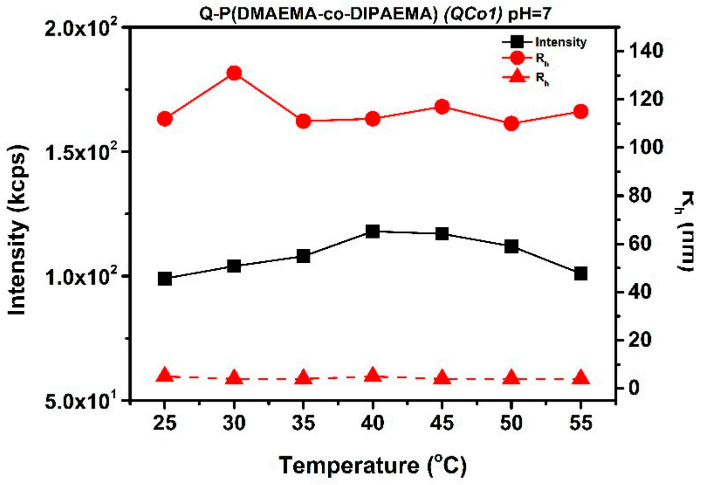

(c)

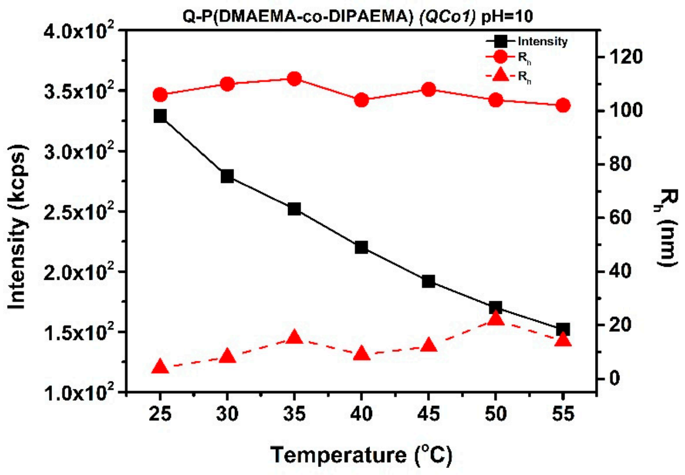

(d)

Figure S7. Size distributions and changes of intensity and hydrodynamic radius as a function of temperature for QP(DMAEMA-co-DIPAEMA) (QCo1) at two different pHs, 7 (**a**, **c** respectively) and 10 (**b**, **d** respectively).

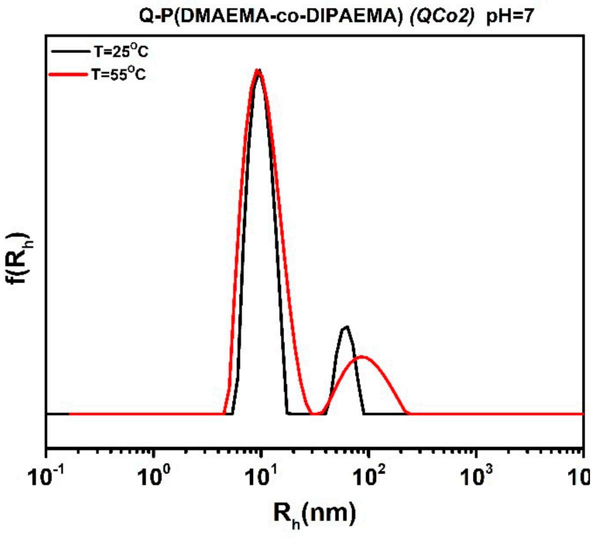

(a)

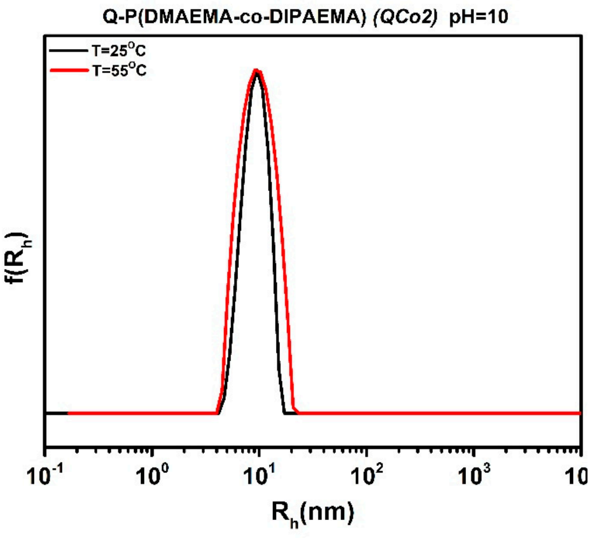

(b)

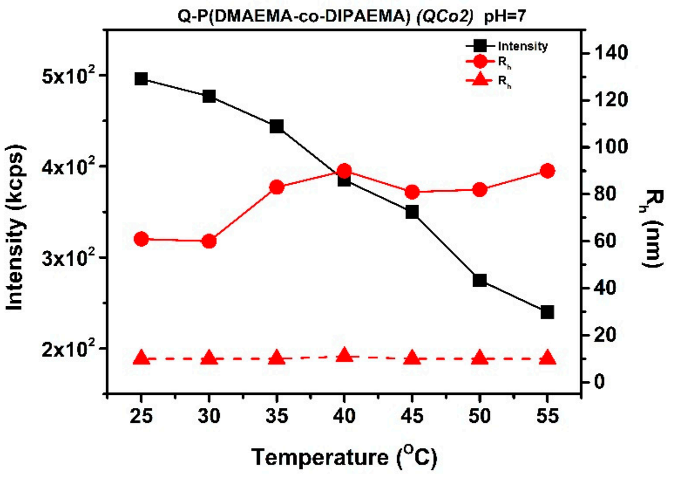

(c)

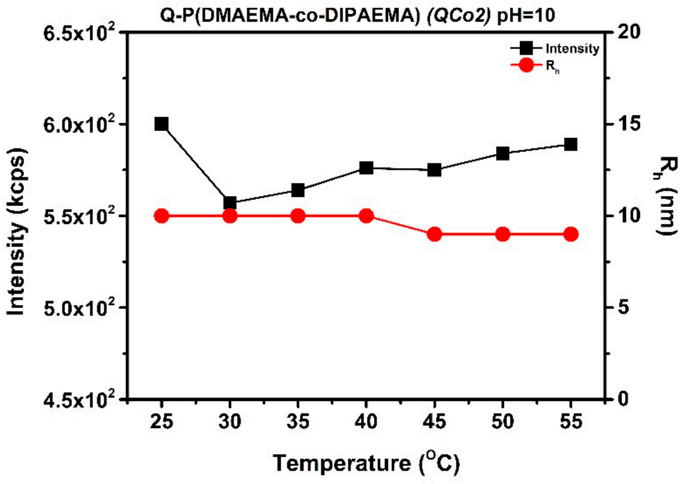

(d)

Figure S8. Size distributions and changes of intensity and hydrodynamic radius as a function of temperature for QP(DMAEMA-co-DIPAEMA) (QCo2) at two different pHs, 7 (**a**, **c** respectively) and 10 (**b**, **d** respectively).

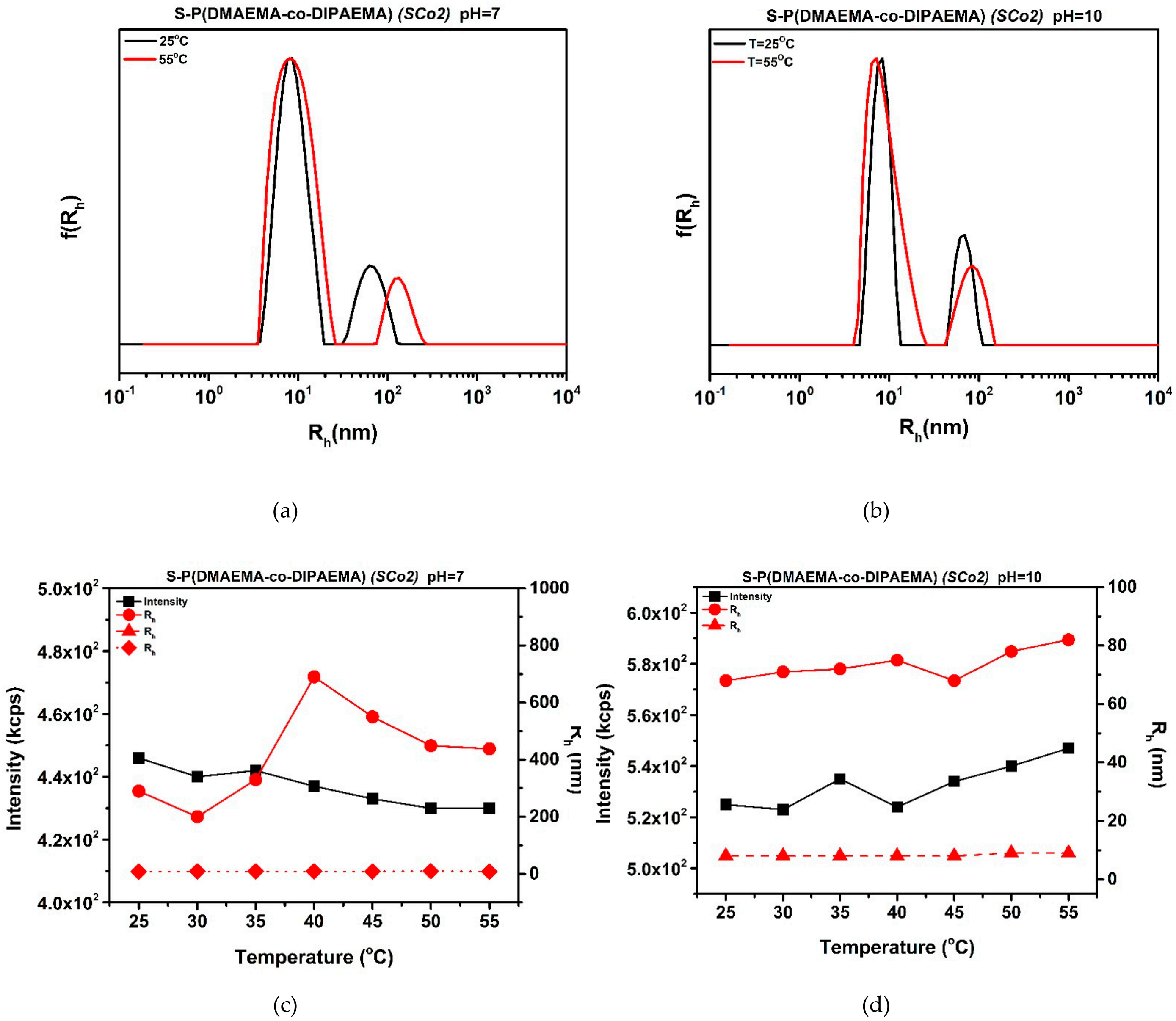

Figure S9. Size distributions and changes of intensity and hydrodynamic radius as a function of temperature for SP(DMAEMA-co-DIPAEMA) (SCo2) at two different pHs, 7 (**a**, **c** respectively) and 10 (**b**, **d** respectively).

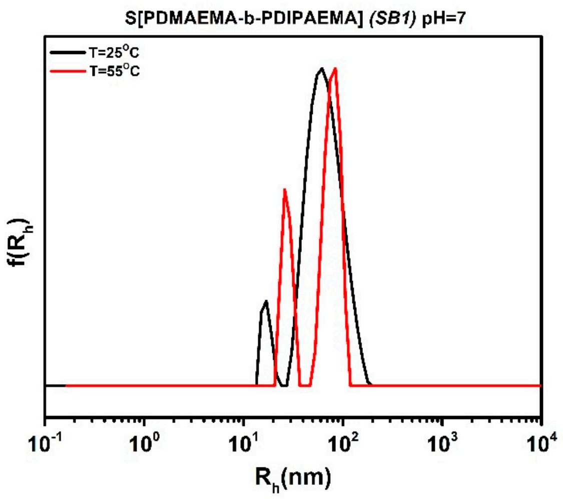

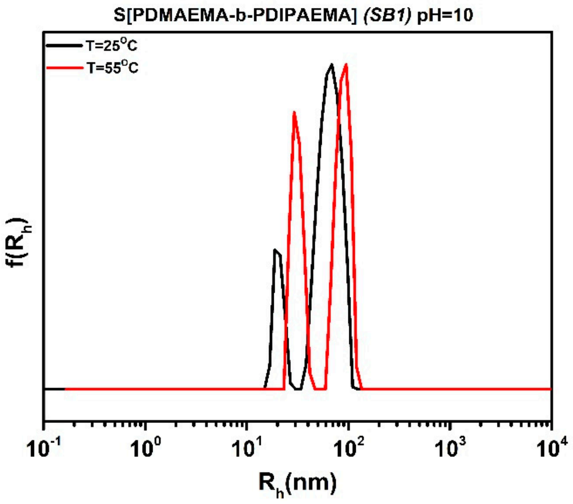

(a) (b)

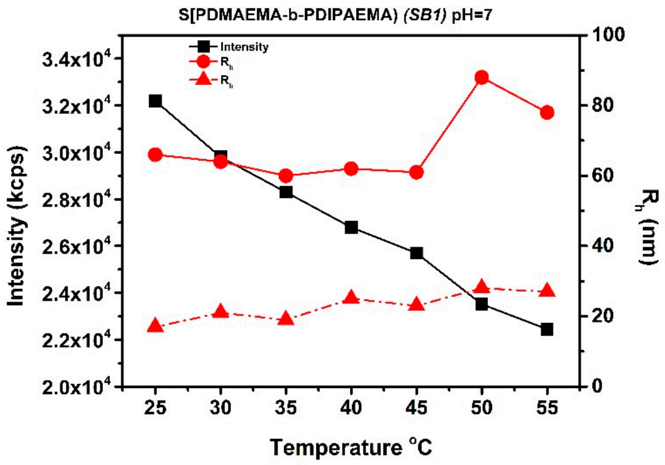

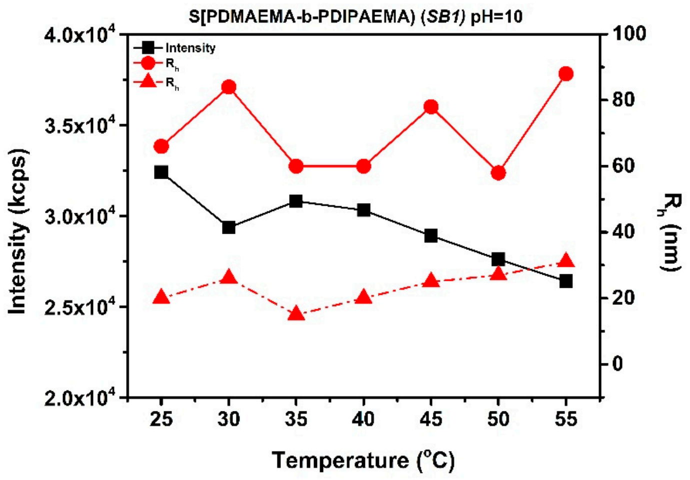

(c) (d)

Figure S10. Size distributions and changes of intensity and hydrodynamic radius as a function of temperature for SPDMAEMA-b-PDIPAEMA (SB1) at two different pHs, 7 (**a**, **c** respectively) and 10 (**b**, **d** respectively).

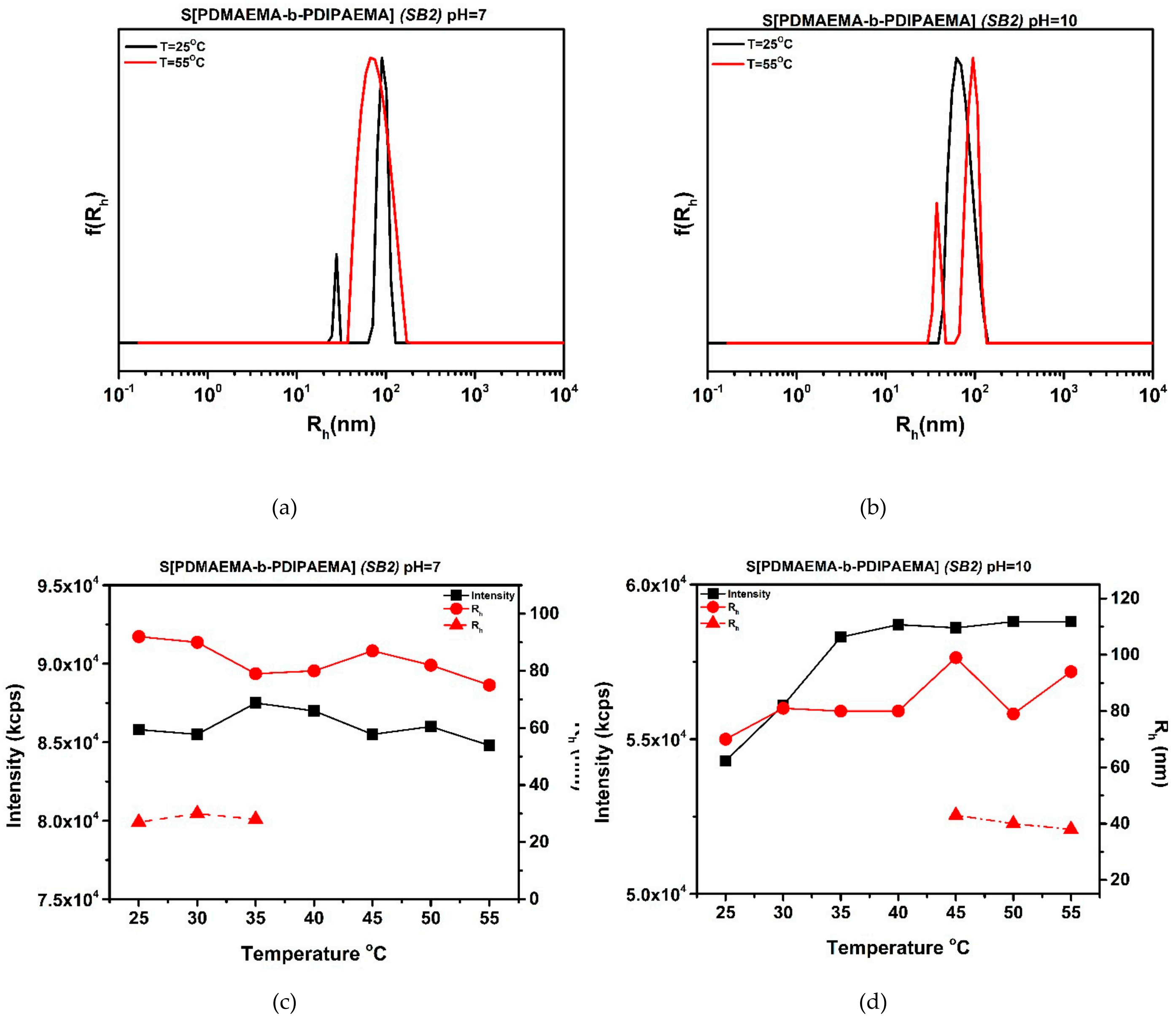

Figure S11. Size distributions and changes of intensity and hydrodynamic radius as a function of temperature for SPDMAEMA-b-PDIPAEMA (SB2) at two different pHs, 7 (**a**, **c** respectively) and 10 (**b**, **d** respectively).

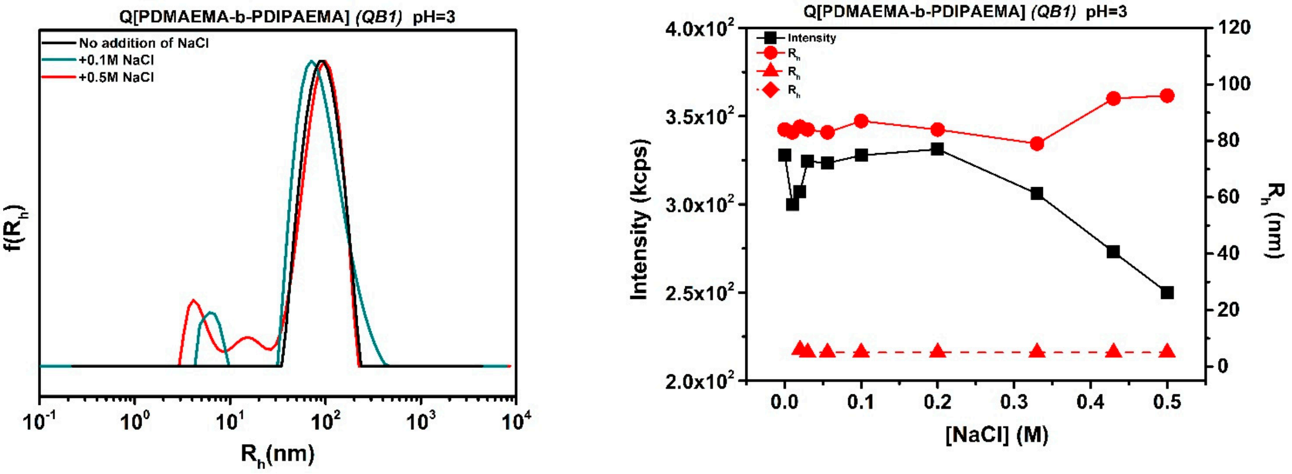

(a) (b)

Figure S12. Size distributions and changes of intensity and R_h as a function of increasing ionic strength for copolymer QPDMAEMA-b-PDIPAEMA (QB1) aqueous solutions.

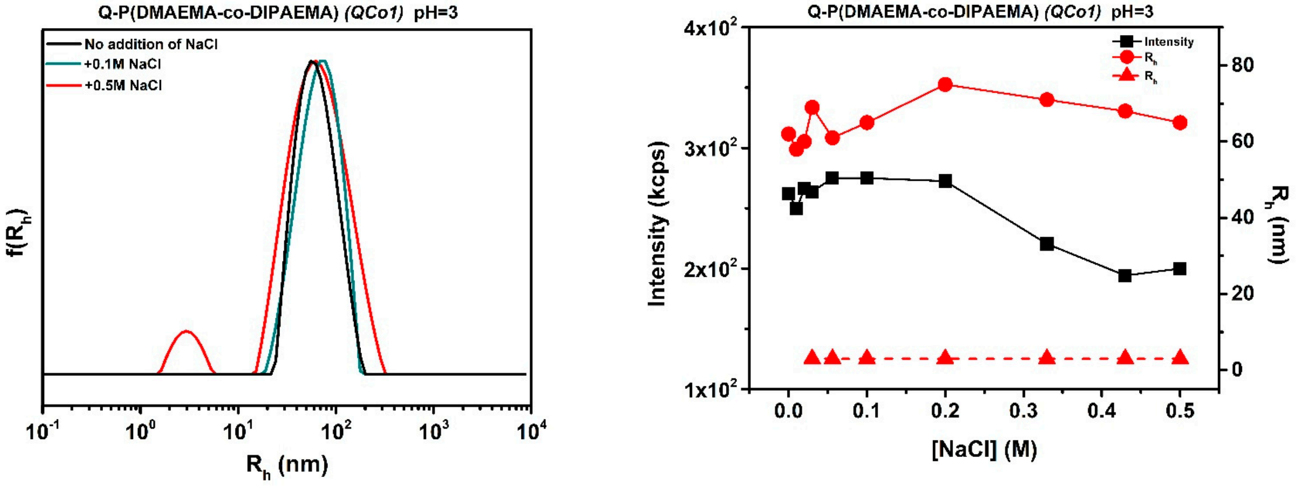

(a) (b)

Figure S13. Size distributions and changes of intensity and R_h as a function of increasing ionic strength for copolymer QP(DMAEMA-co-DIPAEMA) (QCo1) aqueous solutions.

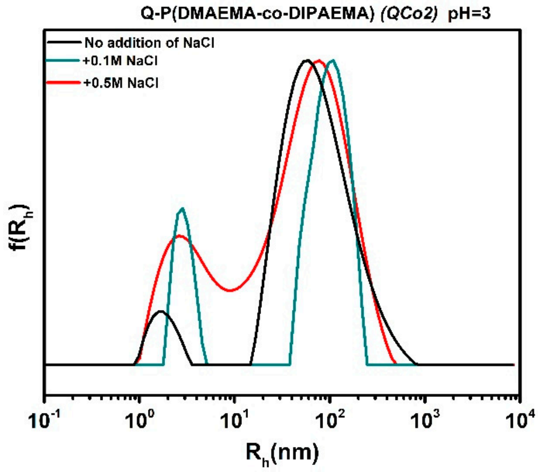

(a)

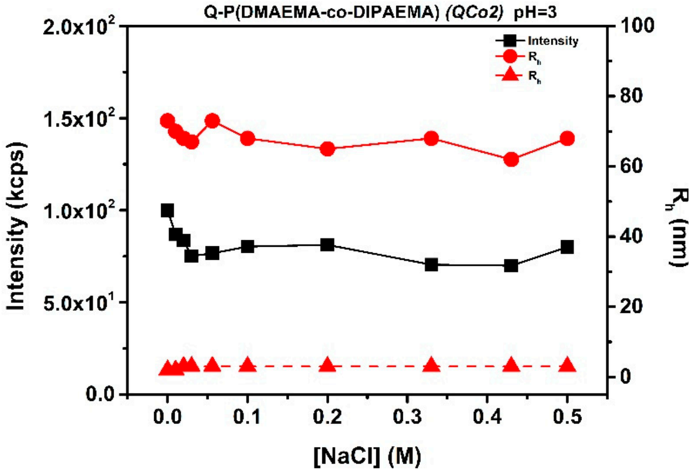

(b)

Figure S14. Size distributions and changes of intensity and Rh as a function of increasing ionic strength for copolymer QP(DMAEMA-co-DIPAEMA) (QCo2) aqueous solutions.

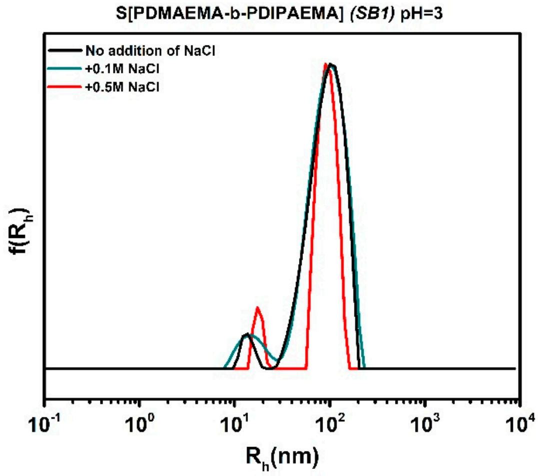

(a)

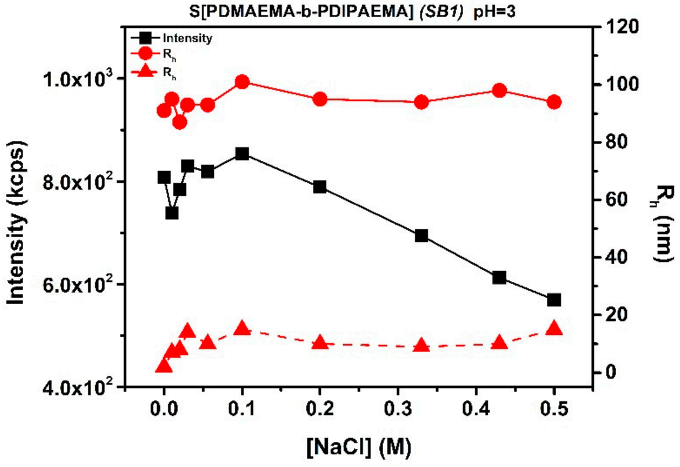

(b)

Figure S15. Size distributions and changes of intensity and Rh as a function of increasing ionic strength for copolymer SPDMAEMA-b-PDIPAEMA (SB1) aqueous solutions.

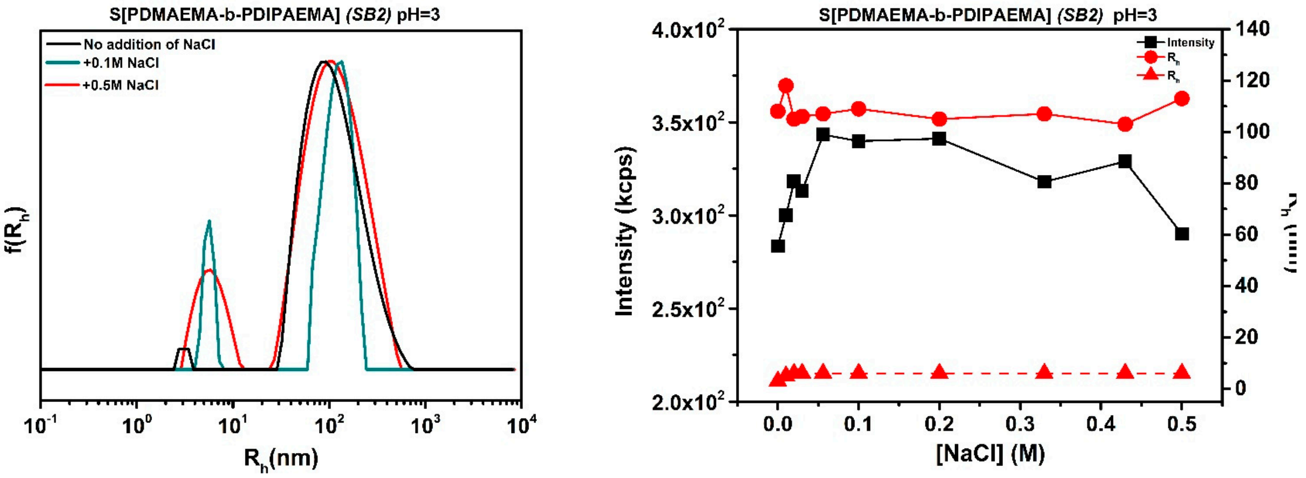

(a) (b)

Figure S16. Size distributions and changes of intensity and Rh as a function of increasing ionic strength for copolymer SPDMAEMA-**b**-PDIPAEMA (SB2) aqueous solutions.

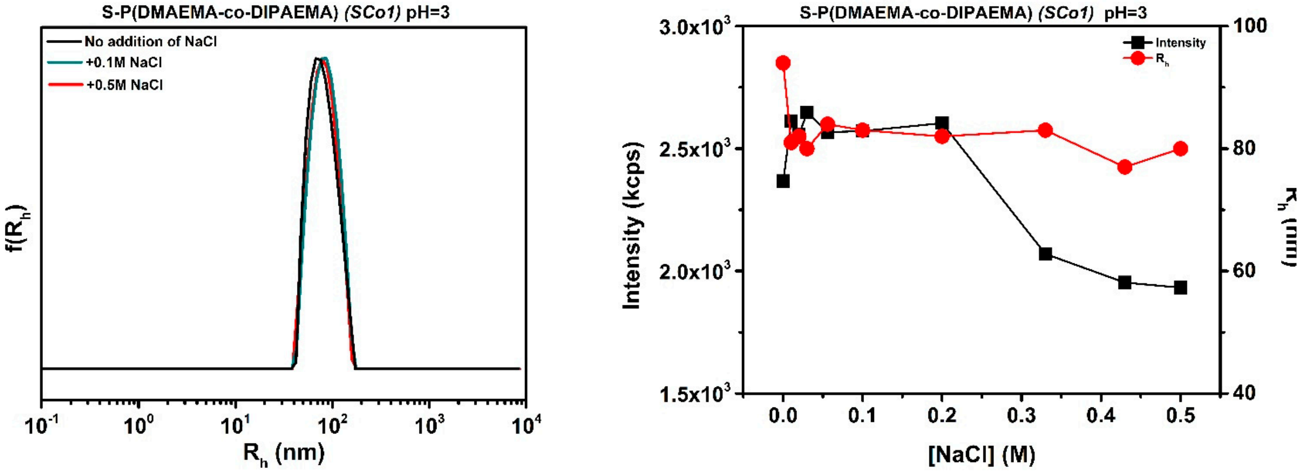

(a) (b)

Figure S17. Size distributions and changes of intensity and Rh as a function of increasing ionic strength for copolymer SP(DMAEMA-co-DIPAEMA) (SCo1) aqueous solutions.
